# Supplementary material for: Characterization of a novel Phietavirus genus bacteriophage and its potential for efficient transfer of modified shuttle plasmids to Staphylococcus aureus strains of different clonal complexes
Source: Microbiol Spectr. 2025 Jul 11;13(8):e03332-24. doi: 10.1128/spectrum.03332-24 (PMC12323379; doi:10.1128/spectrum.03332-24)
Supplement: Supplemental material — Tables S1 to S11, Figures S1 to S7, and supplemental references. [file spectrum.03332-24-s0001.pdf]

Supplementary Tables

Table S1. *Staphylococcus* spp. strains used in this study

Strain	Clonal Complex	MRSA/ MSSA	Sequence type	*spa* type	*agr* type	Source of isolation	Accession number$	Source or reference
S. aureus clinical and environmental strains								
717/05*	CC1	MSSA	ST1847	t127	3	sputum	CP170797; CP170798 (p1)	this study
1203/05*		MSSA	ST1	t607	3	wound swab	CP170693; CP170694 (p1); CP170695 (p2)	this study
1693/05*		MSSA	ST1	t127	3	wound swab	CP170676	this study
3572/09*#		MSSA	ST1847	t786	3	ulce swab	CP170787; CP170788 (p1); CP170789 (p2)	this study
1034/05*	CC5	MRSA	ST496	t002	2	blood	CP170683; CP170684 (p1)	this study
1072/05#		MSSA	ST5	t053	2	wound swab	NA	this study
1793/05*		MRSA	ST496	t002	2	blood	CP170799	this study
1880/05*		MRSA	ST5	t458	2	wound swab	CP170707; CP170708 (p1); CP170709 (p2)	this study
2875/05*		MRSA	ST228	t041	2	blood	CP170605; CP170606 (p1)	this study
300/07*		MRSA	ST225	t003	2	blood	CP170783; CP170784 (p1)	this study
3381/07#		MRSA	ST5	t283	2	blood	NA	this study
4000/07*		MRSA	ST5	t053	2	pus	CP170790; CP170791 (p1); CP170792 (p2)	this study
10250/11*		MRSA	ST5	t777	2	blood	CP170813	this study
1171/05*#	CC7	MSSA	ST1604	t091	1	wound swab	CP170691; CP170692 (p1)	this study
1268/05*#		MSSA	ST7	t091	1	blood	CP170696; CP170697 (p1)	this study
4472/08*#		MRSA	ST7	t091	1	blood	CP170439; CP170440 (p1)	this study
10525/11*#		MRSA	ST3975	t091	1	blood	CP170687; CP170688 (p1)	this study
10174/99*	CC8	MRSA	ST247	t052	1	blood	CP170681; CP170682 (p1)	this study
2065/05*		MSSA	ST8	t052	1	wound swab	CP170771; CP170772 (p1)	this study
2261/05*		MSSA	ST8	t008	1	blood	CP170773; CP170774 (p1); CP170775 (p2); CP170776 (p3); CP170777 (p4)	this study

2945/06*		MRSA	ST247	t052	1	blood	CP170781; CP170782 (p1)	this study
3009/07*		MRSA	ST8	t008	1	blood	CP170785; CP170786 (p1)	this study
10415/11*		MRSA	ST8	t008	1	blood	CP170713; CP170714 (p1); CP170715 (p2)	this study
101/00*	CC8/239	MRSA	ST241	t037	1	blood	CP170677; CP170678 (p1); CP170679 (p2); CP170680 (p3)	this study
2262/05*		MRSA	ST239	t030	1	sputum	CP171380; CP171381 (p1); CP171382 (p2)	this study
4065/07*		MRSA	ST239	t4557	1	blood	CP170764	this study
4124/07*		MRSA	ST239	t037	1	pus	CP170685; CP170686 (p1)	this study
2064/05*	CC9	MSSA	ST9	t2700	2	cerbero-spinal fluid	CP170800	this study
3864/05*		MSSA	ST9	t1313	2	blood	CP170763	this study
1881/05*	CC15	MSSA	ST15	t084	2	blood	CP170769; CP170770 (p1)	this study
1700/07*		MSSA	ST15	t084	2	blood	CP170698; CP170699 (p1); CP170700 (p2)	this study
2929/07*		MSSA	ST15	t084	2	blood	CP170778; CP170779 (p1); CP170780 (p2)	this study
3812/16#		MSSA	ST15	t084	2	environment	NA	this study
706/05*	CC22	MSSA	ST1911	t223	1	wound swab	CP170795; CP170796 (p1)	this study
6068/10*		MRSA	ST22	t852	1	blood	CP170793; CP170794 (p1)	this study
2584/01*	CC30	MRSA	ST30	t391	3	throat swab	CP171451	this study
1528/09*		MRSA	ST36	t018	3	blood	CP170675	this study
4341/10 (PS80)*		MSSA	ST30	t021		unknown	CP171210; CP171211 (p1); CP171212 (p2)	Colindale collection via Janusz Galiński; Asheshof; 1969
1286/05*	CC45	MSSA	ST45	t1081	1	bone swab	CP170673	this study
1452/05*		MSSA	ST45	t620	1	blood	CP170712	this study
1786/05*		MSSA	ST45	t015	1	blood	CP170767; CP170768 (p1)	this study

1105/06*		MRSA	ST45	t050	1	blood	CP170689; CP170690 (p1)	this study
2795/06*		MSSA	ST8142	t015	1	synovial fluid	CP194533	this study
247/07*		MRSA	ST45	t1574	1	blood	CP171383; CP171384 (p1)	this study
140/05*	CC59	MSSA	ST59	t529	1	blood	CP170674	this study
1330/05#		MRSA	ST338	t437	1	wound swab	NA	this study
1781/05*		MRSA	ST338	t1751	1	blood	CP170765; CP170766 (p1)	this study
10497/11#		MSSA	ST59	t216	1	blood	NA	this study
1078/13#	CC97	MSSA	ST97	t521	1	cow's milk	NA	this study
3793/16#		MSSA	ST97	t16317	1	cow's milk	NA	this study
5069/08*	CC398	MRSA	ST398	t108	1	nasal swab	CP170607; CP170608 (p1); CP170609 (p2)	this study
5074/08*		MSSA	ST398	t034	1	nasal swab	CP170610; CP170611 (p1)	this study
463/10*		MRSA	ST398	t034	1	blood	CP170710; CP170711 (p1)	this study
7202/12*		MSSA	ST398	t1928	1	nasal swab	CP170621	this study
S. aureus laboratory strains								
RN4220#	CC8	MSSA	ST8	t211			NZ_CP076105	Nair et al., 2011; Berscheid et al., 2012
80wphwpl_v1#	CC30	MSSA	ST30	t1414		Derivative of PS80	NZ_CP034098	Głowacka-Rutkowska et al., 2019
Other *Staphylococcus* strains								
S. hyicus DSM-17421*	NA	NA	NA	NA		unknown	NA	DSMZ; Devriese et al. , 1978
S. lugdunensis DSM-4804*	NA	NA	NA	NA		axillary lymph node	NA	DSMZ; Freney et al., 1988

# - strains used to prepare the enrichment culture; * - strains used for spot tests, NA - not applicable, $ - (p) -plasmid

Table S2. Dimensions of the ASZ22RN virions

Virion	Tail and tip lenth [nm]	Tail length [nm]	Tail tip length [nm]	Capsid diameter [nm]
1	192	158	34	59
2	162	131	31	62
3	152	120	32	61
4	183	154,5	28,5	60
5	171	143	28	57
6	209	186	23	57
7	181	156	25	54
8	150	125	25	59
9	187	154	33	53
10	165	139	26	48
11	157	134	23	61
12	157	134	23	62
13	160	128	32	58
14	175,5	149,5	26	62
15	191	158	33	57
16	146	122	24	60
17	175	147,5	27,5	53
18	163	134,5	28,5	53
19	162	133,5	28,5	55
20	154	130	24	52
Average	169.63	141.88	27.75	57.15
Standard deviation	16.66	16.11	3.7	3.98

*Dimensions of particular virion parts were measured with the use of 5 electron micrographs, with the scale bar

Table S3. *In silico* analysis of the ASZ22RN genomic sequence with respect to staphylococcal siphoviruses typing markers

Genome module	Typing marker[a]	Module type	Length of PCR product	Exemplary phage representative	GenBank acc. no.	Genus
Lysogeny	integrase	Sa5int	375	Staphylococcus phage phi 11<br>Staphylococcus phage phiMR25<br>Staphylococcus phage 29	NC_004615<br>NC_010808<br>NC_007061	*Dubowvirus*<br>*Dubowvirus*<br>*Phietavirus*
Lysogeny control	antirepressor	ant1a	317	Staphylococcus phage phi 11<br>Staphylococcus phage 42E	NC_004615<br>NC_007052	*Dubowvirus*<br>*Triavirus*
DNA replication	Phage DnaC, subgroup C2	dnaC2	389	Mu50B	NC_002758[b]	unclassified
Transcription regulation	dUTPase	dut2	289	Mu50B<br>Staphylococcus phage phiETA3<br>Staphylococcus phage phi2958PVL<br>Staphylococcus prophage phiPV83<br>Staphylococcus phage phiSauS-IPLA88	NC_002758[b]<br>NC_008799<br>NC_011344<br>NC_002486<br>NC_011614	unclassified<br>*Phietavirus*<br>*Triavirus*<br>*Peeveelvirus*<br>*Dubowvirus*
Morphogenesis (packaging)	portal	Bb	453	Staphylococcus phage 96<br>Staphylococcus phage phinm4	NC_007057<br>DQ530362	*Phietavirus*<br>*Phietavirus*
Morphogenesis (tail appendices)	Phage type (tail appendices)	serogroup B-like phages	405	Staphylococcus phage phi 11<br>Staphylococcus phage phiETA3	NC_004615<br>NC_008799	*Dubowvirus*<br>*Phietavirus*
Lytic	amidase	ami1	364	Staphylococcus phage phi 11<br>Staphylococcus phage 55	NC_004615<br>NC_007060	*Dubowvirus*<br>*Phietavirus*

[a]Typing was performed according to Kahankova et al., 2010 (6).

[b]The accession number of Mu50B genomic sequence applies to the accession number of *S. aureus* strain in which Mu50B was identified as a prophage.

Table S4. Results of HHpred analysis of ASZ22RN proteins. The best and most relevant hits are shown, including the coordinates of the matching regions, PDB ID and chain identifier, the HHpred probability (%) in the matched regions, and the identity (%). Only proteins with HHPred probability results over 70% are presented.

Product of gene:	Protein length (aa)	Matchig region	HHPred best match(es)	PDB ID	Probability (%)	E-value	Sequence Identity (%)
1	164	2-122	*Bacillus* phage SF6<br>Terminase small subunit	3ZQP_D	99.76	2E-17	24
2	407	6-400	*Geobacillus stearothermophilus* phage D6E<br>Terminase large subunit	5OE8_C	100	4.8E-43	25
3	474	2-453	*Bacillus* phage SPP1<br>Portal protein	2JES_K	100	5.6E-42	23
4	317	141-283	Halofax tailed virus<br>Portal protein, portal-capsid interface	8QQN_PL	99.75	5.8E-18	16
6	194	43-163	*Staphylococcus* phage 80alpha<br>Scaffold protein	6B0X_f	99.71	2.1E-15	22
7	304	11-303	*Staphylococcus* phage 80alpha<br>Major head protein	6B0X_G	100	1.4E-31	42
9	116	5-109	*Bacillus* phage SPP1<br>Protein 15, head-to-tail interface	5A21_C	99.74	9.5E-17	26
10	111	4-110	*Bacillus* phage SPP1<br>Head completion protein gp16	5A21_F	99.88	1.2E-20	12
		3-108	*Rhodobacter capsulatus*<br>Stopper protein Rcc01689	6TE9_E	99.84	2.7E-19	9
11	137	1-45	*Methanocaldococcus jannaschii*<br>V-type ATP synthase subunit E	2KK7_A	85.75	20.43	20
12	141	1-132	*Rhodobacter capsulatus*<br>Tail terminator protein Rcc01690	6TE9_F	99.92	3.7E-22	17
		1-333	*Bacillus* phage SPP1<br>Tail-to-head joining protein gp17, DNA gatekeeper	5A21_G	99.89	8E-21	26
		1-135	*Listeria monocytogenes*<br>Prophage LambdaLm01, antigen B	4ACV_B	99.84	7.3E-19	12
		1-132	*Enterobacteria* phage Lambda<br>Minor tail protein U	3FZ2_I	98.78	6E-7	9
13	185	1-167	*Staphylococcus* phage 80alpha<br>Major tail protein gp53	6V8I_FA	99.95	1.5E-26	15
		1-168	*Bacillus* phage SPP1<br>Tail tube protein gp17.1	6YQ5_L	99.92	2.7E-23	22
14	168	83-162	UPF0335 protein ASE63_04290; Caulobacter, DNA binding, NAP, DNA twist,	6CFX_A	79.37	22	13
16	1047	1-953	*Staphylococcus* phage 80alpha<br>Tape measure protein gp57	6V8I_AF	100	1.4E-46	35
17	313	4-313	*Staphylococcus* phage 80alpha<br>Distal tail protein gp58	6V8I_BD	100	1.1E-33	28

18	628	1-628	*Staphylococcus* phage 80alpha Tail-associated lysin gp59	6V8I_CE	100	2.4E-66	61
		414-627	*Sinorhizobium meliloti* Hydrolase	5HOE_B	99.69	6.9E-16	13
		415-623	*Streptomyces rimosus* Lipase	5MAL_A	99.65	7.3E-16	15
		1-327	*Neisseria meningitidis* Tail protein	3D37_A	99.24	3.4E-8	11
19	632	59-628	*Staphylococcus* phage P68 Tail fiber	6IAB_A	100	3.7E-86	26
20	607	1-607	*Staphylococcus* phage 80alpha Lower fiber gp62,	6V8I_CJ	100	6.7E-81	95
		388-608	*Staphylococcus* phage K Putative receptor-binding protein	5M9F_A	99.79	9E-18	18
		10-167	*Lactococcus* phage TP901-1 Distal tail protein	4V96_AQ	99.61	1.1E-13	19
21	125	93-125	De novo designed protein coiled-coil peptide	4DZN_A	88.08	1.1	10
22	58	3-52	*Bacillus* phage SPP1 gp23.1 putative chaperone	2XF7_A	99.83	3.7E-20	14
		23-43	Influenza A virus RNA-directed RNA polymerase catalytic subunit	3A1G_A	85.55	1.7	29
		22-42	*Streptococcus pneumoniae* Antilipopolysaccharide factor	2JOB_A	79.87	3.1	14
23	99	6-65	*Enterobacteria* phage P22 Tail needle protein gp26	4ZXQ_D	96.22	0.13	10
		6-65	*Enterobacteria* phage HK620 DNA stabilization protein	5BU5_D	95.97	0.16	8
		73-97	*Enterococcus faecalis* Putative uncharacterized protein RNAI, toxin-antitoxin, toxin	2KV5_A	79.88	5.4	24
		77-97	*Staphylococcus aureus* Toxin	4B19_A	71.42	12	24
24	624	397-624	*Staphylococcus aureus* LYZ2 domain-containing protein, peptidoglycan hydrolase	6U0O_B	99.97	7.3E-31	39
		398-624	*Staphylococcus aureus* Bifunctional autolysin, peptidoglycan hydrolase	6FXO_A	99.97	1.5E-29	42
		10-191	*Enterococcus* phage IMEEF1 Lysin, hydrolase	6IST_C	99.59	1.6E-13	26
		209-321	*Streptococcus pneumoniae* Autolysin, amidase	4X36_A	98.63	2E-7	7
25	412	1-412	*Staphylococcus* phage 80alpha Upper fiber	6V8I_AM	100	3.8E-30	89
26	131	56-114	*Drosophila melanogaster* FK506-binding nuclear protein	4CA9_B	74.31	18	22
		29-77	*Homo sapiens* Fibronecrin	4GH7_B	73.34	13	20
		49-118	*Mus musculus* Cytokine receptor-like factor 3	6RPX_A	72.52	23	15

27	145	5-93	Uncharacterized membrane protein Cgl2017/cg2211	7Q21_H	77.17	9.3	17
28	481	176-366	*Staphylococcus* phage G15 Endolysin	4OLS_A	99.84	1.3E-18	53
		197-362	*Staphylococcus aureus* Bifunctional autolysin	4KNK_A	99.56	3.3E-13	20
		28-154	*Escherichia coli* Bifunctional glutathionylspermidine synthetase/amidase	3A2Z_A	99.2	3E-10	15
		387-481	*Staphylococcus simulans* Lysostaphin	6RK4_A	98.55	4.3E-7	58
32	348	59-344	*Escherichia coli* Site-specific recombinase	1A0P_A	100	2.3E-32	17
		55-347	*Escherichia* phage P2 Integrase	5C6K_B	100	3.6E-30	16
		1-343	*Escherichia* phage lambda Integrase	1Z1B_B	99.97	6.3E-29	17
33	156	53-154	*Korarchaeum cryptofilum* Uncharacterized protein; coiled coil, beta-layer	6H9L_A	99.16	2.5E-9	11
		57-139	*Homo sapiens* Apolipoprotein A-I	1AV1_D	93.99	2	11
		57-153	*Serratia marcescens* SmhA, pore forming toxin	7A27_AAA	93.30	4.9	12
		56-154	*Serratia marcescens* SmhB, pore forming toxin	6ZZ5_AAA	89.39	14	12
		54-163	*Aeromonas hydrophula* AhlB pore-forming toxin	6GRK_B	93.11	5.7	14
		55-161	*Aeromonas hydrophila* AhlC pore-forming toxin	6R1J_D	81.65	31	17
		54-159	*Bacillus cereus* toxin hemolysin BL lytic component	7NMQ_A	91.84	4	8
		54-162	*Bacillus cereus* pore-forming toxin, NheA component	4K1P_C	89.82	13	11
		54-163	*Vibrio cholerae* tripartite toxin component	6DFP_A	92.64	6.7	17
35	44	8-43	*Geobacillus thermodenitrificans* Putative mambrane protein, bd oxidase	5DOQ_C	77.88	7.4	14
36	153	3-127	*Deinococcus deserti* IrrE protein	3DTE_A	99.63	1.4E-14	18
		13-127	*Mycobacterium tuberculosis* HTH-type transcriptional	6D2S_A	99.56	3E-13	14

			regulator PrpR				
		13-127	*Saccharomyces mikatae* Membrane protein alpha helical, CaaX protease, a-factor	4IL3_B	96.54	0.036	14
37	107	1-102	*Streptococcus vestibularis* Transcriptional regulator ComR	6HU8_A	99.19	8.2E-9	19
		7-102	*Citrobacter sp.* Csp231I C protein; helix-turn-helix, C controller protein, restriction-modification systems	4JCY_A	99.00	8.9E-8	20
		1-100	*Staphylococcus aureus* SaPI repressor	6H49_A	98.81	1.1E-6	19
38	82	4-68	*Homo sapiens* Endothelial differentiation-related factor 1	1X57_A	98.59	2.2E-6	23
		3-67	*Streptococcus vestibularis* Transcriptional regulator ComR	6HU8_A	98.55	3.6E-6	17
		3-68	*Staphylococcus aureus* SaPI repressor	6H49_A	98.48	9E-6	20
39	147	93-125	*Birnavirus* (IBDV) Capsid assembly protein VP3; helix, Capsid protein, Hydrolase, Protease, Serine protease	2R18_A	64.18	22	21
40	59	4-36	*Escherichia coli* 50S ribosomal protein L7/L12	1RQT_B	92.84	0.38	18
		11-52	*Homo sapiens* Ubiquitin-like protein 4A	4X86_A	84.58	4.7	18
42	263	160-194	*Sulfolobus islandicus* DNA binding protein	7BZH_A	74.47	15	14
45	71	2-59	*Streptomyces venezuelae* Putative DNA-binding protein BldC	6AMA_A	99.03	4.2E-10	13
		2-56	*Enterobacteria* phage P2 Regulatory protein cox	4LHF_A	98.93	6.3E-10	11
		3-57	*Enterococcus faecalis* Repressor, protein-DNA complex, type IV secretion system	6G1T_A	98.74	1.2E-8	15
		1-58	*Escherichia coli* Redirecting phage packing protein C RppC	6HLK_A	98.52	1E-7	11
		1-69	*Escherichia coli* Terminase small subunit	6HN7_B	98.34	1.3E-6	12
		1-59	*Enterococcus faecalis* Excisionase from transposon Tn916	1Y6U_A	98.22	1.5E-6	14
47	100	3-27	*Staphylococcus aureus* 50S ribosomal protein L29, methicilin resistance	5T7V_LB	79.50	5.3	28
48	86	31-84	*Myxococcus xanthus* DNA gyrase, B subunit	3CWV_A	67.44	23	7
		31-85	*Francisella tularensis* Topoisomerase IV, subunit B	4HY1_A	65.02	15	20

50	177	18-177	*Desulfovibrio vulgaris* Putative Host-nuclease inhibitor protein Gam	2P2U_B	99.94	1.5E-25	15
51	259	15-205	*Homo sapiens* Meiotic recombination protein DMC1/LIM15 homolog; Recombinase	6R3P_B	99.38	1.1E-11	12
		14-206	*Mycobacterium tuberculosis* Protein RecA	4PPF_A	99.34	4.8E-11	15
		13-204	*Pyrococcus furiosus* DNA repair and recombination protein rad51	1PZN_G	99.33	1E-10	8
52	184	156-184	*Lactococcus* phage P2 Single-stranded DNA binding protein SSB	2WKD_A	95.91	6.5E-3	32
		2-184	*Thermotoga maritima* Single-strand binding protein	1Z9F_A	88.99	2E-2	13
53	230	40-128	*Echerichia* phage Lambda Protein NinB	1PC6_A	99.48	5E-13	24
		132-217	*Enterobacteria* phage P1 Recombination enhancement function protein, HNH nuclease	3PLW_A	98.62	3.7E-8	21
		136-192	*Lactococcus lactis* Group II intron-encoded protein LTRA	5G2X_C	96.46	2.7E-3	19
		133-189	*Escherichia coli* 5-methylcytosine-specific restriction enzyme A, HNH endonuclease	6GHC_A	95.73	1.9E-2	25
		129-204	*Thermosynechococcus vestitus* Maturase reverse transcriptase	6ME0_C	94.53	5E-2	11
54	277	1-90	*Staphylococcus aureus* Replication initiator protein	4PTA_D	96.02	7.7E-3	11
55	261	77-255	*Geobacillus stearothermophilus* Primosomal protein DnaI	4M4W_N	99.89	1.2E-20	20
		70-258	*Escherichia coli* DNA replication protein DnaC	6QEM_J	99.81	1.4E-16	26
56	52	1-52	*Staphylococcus aureus* Phage inhibitor protein	5HE9_E	99.98	9.1E-33	94
58	134	15-110	*Escherichia* phage T7 Endodeoxyribonuclease I, Holliday junction resolvase	1M0D_D	99.32	3.5E-11	21
		14-111	*Escherichia coli* Transposition protein tnsA	1T0F_B	99.08	4.1E-10	17
		6-98	*Escherichia coli Restriction endonuclease EcoP15I, restriction subunit*	4ZCF_C	98.25	3.6E-6	14
		16-111	*Sulfurisphaera tokodaii Endonuclease Bax1*	6P4O_F	97.23	5.5E-3	19
60	205	13-109	*Enterococcus faecalis* AM32; Repressor, Protein-DNA complex, Type IV secretion system, DNA BINDING PROTEIN	6G1T_A	96.62	0.0055	9

62	66	1-52	Recombination protein bet; protein-protein complex, HYDROLASE; HET: SO4; 2.3A {Escherichia phage lambda}	6M9K_D	93.55	0.39	10
64	68	1-68	SGNH_hydro domain-containing protein; phage tail, portal, receptor-binding protein, VIRUS; {Staphylococcus phage Andhra}	8EGR_L	99.78	1.3E-18	36
65	134	1-134	SGNH_hydro domain-containing protein; phage tail, portal, receptor-binding protein, VIRUS; {Staphylococcus phage Andhra}	8EGR_L	100	2.1E-32	31
66	115	1-114	*Staphylococcus aureus* phage *37* hypothetical protein, ORF041,	2P84_A	99.95	7.9E-27	27
		1-114	*Enterococcus faecalis* hypothetical protein	2OX7_D	99.94	9.7E-26	34
		1-114	*Clostridium tetan*i E88 uncharacterized protein	2QYZ_A	99.94	1.3E-25	33
		1-115	YopX protein; NESG, SR411, yopX, O34401, UNKNOWN FUNCTION; HET: MSE; 2.8A *{Bacillus subtilis}*	2I2L_A	99.88	1.2E-21	20
67	129	9-66	*Bacillus subtilis* Initiation-control protein YabA	5DOL_B	82.55	11	18
69	178	1-178	*Staphylococcus aureus* dUTPase	5MYF_A	99.97	2.6E-29	58
70	68	1-46	HYPOTHETICAL PROTEIN HI0828; HI0828, YCII_HAEIN	1MWQ_A	89.66	3.3	13
		1-47	AcrIE2; Anti-CRISPR protein, VIRAL PROTEIN; 1.23A {Pseudomonas phage JBD88a}	8HEK_A	66.4	15	29
71	128	15-106	*Pyrobaculum arsenaticum* pilin	6W8U_V	81.99	16	9
		1-104	*Thermus thermophilus* Cytochrome c oxidase subunit 2	3S8G_B	72.69	21	10
		11-55	*Homo sapiens Calcium-activated potassium channel subunit beta-4*	6V22_F	70.15	21	11
73	133	72-131	*Escherichia coli* RNA polymerase Sigma E factor	1.2H27_D	98.03	0.00019	14
		72-131	*Pseudomonas aeruginosa* PAO1 RNA polymerase sigma-H factor;	6IN7_B	98.00	0.00016	19
		19-132	Streptomyces venezuelae strain ATCC 10712 RNA polymerase sigma factor; sigma, anti-sigma, c-di-GMP, developmental switch	6PFJ_A	97.92	0.00035	8

Table S5. Changes in gene annotations introduced in the phage genomes that have been used for the alignment shown in Figure 3.

Phage (GeneBank Acc, No.)	Removed genes	Added genes	Shortened genes		Elongated genes	
			Old coordinates	New coordinates	Old coordinates	New coordinates
3MRA (NC028917)	YP_ 009209255.1<br>YP_ 009209305.1<br>YP_009209295.1	18,144-18,644<br>17,059-17,160<br>3,613-3,765<br>41,775-41,900<br>40,152-40,241<br>39,994-40,095<br>39,772-39,892<br>39,527-39,649<br>37,119-37,253<br>35,865-36,101<br>33,072-33,233<br>22,997-23,089	40,270-41,742<br>32,413-32,739	40,270-41,715<br>32,413-32,673	22,378-22,545<br>16,356-16,574<br>14,962-15,357<br>14,393-14,758<br>14,064-14,213<br>10,219-10,575<br>611-1,666<br>27,951-28,583	22,378-22,872<br>16,356-16,691*<br>14,962-15,518<br>14,393-14,899<br>14,064-14,348<br>10,219-11,160<br>429-1,666*<br>27,951-28,736
96 (NC_007057	None	8,912-9,196<br>25,542-25,685<br>25,717-25,809<br>25,862-26,017<br>26,459-26,569<br>26,652-26,780<br>4-159	6,554-6,898<br>9,200-12,343	9,215-12,343<br>17,210-17,671	3,261-4,214<br>8,502-8,867<br>12,764-13,299	3,246-4,214<br>8,361-8,867<br>12-358-13,299
phiJB (NC_028669)	ALA12219.1<br>ALA12160.1	42,459-42,626<br>42,672-42,764<br>1,268-1,375<br>16,962-17,054<br>17,062-17,175	17,177-17,671<br>26,216-29,359	17,210-17,671<br>26,231-29,359	20,277-21,230<br>23,901-24,314<br>3,010-3,324	20,262-21,230<br>23,865-24,314<br>3,010-3,582
Mh1 (OM439673)	UMK36479.1	42,640-42,735<br>42,867-42,959<br>43,012-43,167<br>43,608-43,718<br>2,743-2,898<br>4,949-5,110<br>17,197-17,289<br>17,286-17,411	23,444-23,803<br>23,809-24,150<br>26,470-29,355<br>8,072-8,731	23,453-23,803<br>23,815-24,150<br>26,485-29,355<br>8,072-8,722	None	
DW2 (NC_024391)	YP_ 009045043.1<br>YP_009045002.1<br>YP_ 009045020.1	23,843-23,944<br>23,931-24,080<br>34,842-34,964<br>35,051-35,200<br>35,379-37,073<br>41,078-41,260<br>41,267-41,377<br>41,370-41,498<br>41,537-41,728	15,045-15,539<br>24,084-27,227<br>34,192-34,368<br>10,975-11,232	15,078-15,539<br>24.099-27,227<br>34,195-34,368<br>10,984-11,232	18,145-19,098<br>20,936-21,079<br>39,140-39,577<br>11,247-11,453	18,130-19,098<br>20,933-21,079<br>39,029-39,577<br>11,244-11,453

		41,774-41,893<br>4,434-4,526<br>11,881-12,003<br>14,758-14,874<br>14,972-15,043				
phiETA3 (NC_008799)	None	43,206-52<br>12,709-12,810<br>16,870-16,986<br>17,039-17,155	26,043-26,192<br>4,327-4,521	25,908-26,192<br>4,207-4,521	26,043-26,192<br>4,327-4,521	25,908-26,192<br>4,207-4,521
B166 (NC_028859)	None	2,972-3,064<br>9,160-9,264<br>15,560-15,658<br>16,766-16,858	16,982-17,476<br>25,467-25,790<br>25,794-28,679<br>35,644-35,820<br>40,132-41,553<br>11,411-11,827<br>11,831-12,208<br>14,637-15,191<br>15,663-16,058	17,015-17,476<br>25,641-25,790<br>25,809-28,679<br>35,647-35,820<br>40,141-41,553<br>11,423-11,827<br>11,840-12,208<br>14,655-15,191<br>15,672-16,058	None	
B122 (NC_054979)	YP_010079826.1	36,313-36,465<br>1,118-1,231<br>16,991-17,083	17,208-17,702<br>26,023-28,908	17,241-17,702<br>26,038-28,908	18,921-20,339<br>4.,328-4,522<br>9,193-9,963	18,915-20,339<br>4,208-4,522<br>9,103-9,963
55 (NC_007060)	YP_ 240456.1<br>YP_ 240457.1<br>YP_ 240464.1<br>YP_ 240470.1<br>YP_ 240475.1<br>YP_ 240481.1<br>YP_ 240485.1<br>YP_240493.1<br>YP_ 240502.1<br>YP_ 240509.1<br>YP_ 240510.1<br>YP_ 240528.1	174-263<br>25,081-25,170<br>25,319-25,447<br>29,645-29,779<br>30,538-30,669<br>30,737-30,844<br>41,659-41,748<br>28-141	6,700-7,113	6,763-7,113	1,852-3,270<br>25,630-25,815<br>29,804-30,052<br>5,814-5,942	1,792-3,270<br>25,615-25,815<br>29,780-30,052<br>5,805-5,942
80alpha (NC_009526)	YP_ 001285371.1<br>YP_ 001285374.1<br>YP_ 001285377.1<br>YP_ 001285386.1<br>YP_ 001285316.1<br>YP_ 001285327.1	42,353-42,520<br>42,521-42,649<br>43,583-43,714<br>1,395-1,541<br>2,815-2,928<br>5,973-6,164<br>8,408-8,524	19,545-19,730<br>19,664-20,659<br>13,008-13,355	19,641-19,730<br>19,775-20,659<br>13,011-13,355	15,071-15,457	15,068-15,457

*Corrected genome coordinates of genes encoding essential phage proteins that were absent from original annotations due to sequencing errors

Table S6. DNA content in the phage ASZ22RN transducing particles as detected based on the analysis of Nanopore reads

Name of file with reads	ASZ22RN propagator strain	Number of reads	pMLE5 matches	pLKA18 matches	RN4220 matches [%]
ASZ22-1-ER_ont-1kb-q10.fastq	RN4220/pMLE5	15965	1	NA	28
ASZ22-1-ER_ont-42kb-q10.fastq	RN4220/pMLE5	983	0	NA	0
ASZ22-1-F_ont-1kb-q10.fastq	RN4220/pMLE5	22693	1	NA	32
ASZ22-1-F_ont-42kb-q10.fastq	RN4220/pMLE5	2019	0	NA	1
ASZ22-2-F_ont-1kb-q10.fastq	RN4220/pLKA18	25423	NA	86	28
ASZ22-2-F_ont-42kb-q10.fastq	RN4220/pLKA18	3123	NA	1	1
ASZ22-2_ont-1kb-q10_run2.fastq	RN4220/pLKA18	110577	NA	391	170
ASZ22-2_ont-42kb-q10_run2.fastq	RN4220/pLKA18	9477	NA	16/19	8
ALL Nanopore reads		190260	2	497	264 [0.14]

Table S7. DNA content in the phage ASZ22RN transducing particles as detected based on the analysis of Illumina reads[1]

Names of files with reads	ASZ22RN propagator strain	Number of paired reads	pMLE5 matches [%]	pLKA18 matches [%]	RN4220 matches [%]
FAG-ASZ22-R1 + R2.fastq	RN4220/pMLE5	6 496 020	18	NA	7 122 [0.11]
FAG-ASZ22-1_trim_R1+R2.fastq1	RN4220/pMLE5	1 216 478	1	NA	1 413 [0.12]
FAG-ASZ22-1_trim_R1+R2.fastq2	RN4220/pMLE5	39 184	0	NA	42 [0.11]
FAG_ASZ22-2_trim_R1+R2.fastq	RN4220/pLKA18	1 123 616	NA	849 (900)[2]	897 [0.08]
FAG-ASZ22-2_5000000_trim_R1+R2.fastq	RN4220/pLKA18	40 652	NA	34 (36)[2]	44 [0.11]
ALL Ilumina reads	RN4220/pMLE5+pLKA18	8 915 950	NA	NA	9 518 [0.11]

[1]The calculations concern the number of paired reads

[2]To avoid including in the calculations matches to *terS*, which would mostly represent ASZ22RN DNA, the search was performed with pLKA18 with the *terS* sequence removed, and thus the result can be underestimated by about 6% (*terS* represents 6% of the pLKA18 sequence), giving in total 936 reads, which represents 0.08% of the total reads.

Table S8. *Staphylococcus aureus* strains containing prophages with the *immIR-cro* intergenic region identical or nearly identical to that of ASZ22RN*

Description	Max Score	Total Score	Query Cover	E. value	Per. Ident	Acc. Len	Accession
S. aureus str. Guangzhou-SAU749 chromosome	295	295	100%	1e-77	100.00%	2840643	CP053185
S. aureus strain UP_551 chromosome	295	295	100%	1e-77	100.00%	2840057	CP047794
S. aureus strain UP_1632 chromosome	295	295	100%	1e-77	100.00%	2806864	CP047777
S. aureus strain ER02878.3 chromosome	295	295	100%	1e-77	100.00%	2968616	CP030713
S. aureus strain ER01422.3 chromosome	295	295	100%	1e-77	100.00%	2908097	CP030692
S. aureus strain ER04181.3 chromosome	295	295	100%	1e-77	100.00%	2907452	CP030547
S. aureus strain ER03864.3 chromosome	295	295	100%	1e-77	100.00%	2873608	CP030566
S. aureus strain ER04174.3 chromosome	295	295	100%	1e-77	100.00%	2877642	CP030525
S. aureus strain ER02988.3 chromosome	295	295	100%	1e-77	100.00%	2828341	CP030588
S. aureus strain ER01935.3 chromosome	295	295	100%	1e-77	100.00%	2870160	CP030540
S. aureus strain ER04164.3 chromosome	295	295	100%	1e-77	100.00%	2837559	CP030542
S. aureus strain ER01009.3 chromosome	295	295	100%	1e-77	100.00%	2852702	CP030489
S. aureus strain ER03444.3 chromosome	295	295	100%	1e-77	100.00%	2911432	CP030475
S. aureus strain ER00551.3 chromosome	295	295	100%	1e-77	100.00%	2900663	CP030424
S. aureus strain ER04219.3 chromosome	295	295	100%	1e-77	100.00%	2908316	CP030404
S. aureus strain ER04567.3 chromosome	295	295	100%	1e-77	100.00%	2865586	CP030411
S. aureus strain 16445 chromosome	295	295	100%	1e-77	100.00%	2940576	CP043302
S. aureus strain 628 chromosome	295	295	100%	1e-77	100.00%	2911773	CP022905
S. aureus strain 545 chromosome	295	295	100%	1e-77	100.00%	2935463	CP022908
S. aureus strain 629 chromosome	295	295	100%	1e-77	100.00%	2913334	CP022904
S. aureus strain 187 chromosome	295	295	100%	1e-77	100.00%	2913163	CP022903
S. aureus strain 191 chromosome	295	295	100%	1e-77	100.00%	2911744	CP022894
S. aureus strain 61 chromosome	295	295	100%	1e-77	100.00%	2911744	CP022893
S. aureus isol. 22_LA_562, chromosome	295	295	100%	1e-77	100.00%	2875548	LT992477
S. aureus isolate 17_LA_343, chromosome	295	295	100%	1e-77	100.00%	2922778	LT992471
S. aureus strain MRSA107 chromosome	295	295	100%	1e-77	100.00%	3095697	CP018629
S. aureus strain CFSAN007847 chromosome	295	295	100%	1e-77	100.00%	2857060	CP017684
S. aureus strain IPLA15 chromosome	295	295	100%	1e-77	100.00%	2781582	CP134618
S. aureus str. UCI 28 isol. ST5 chromosome	295	295	100%	1e-77	100.00%	2835307	CP018768
S. aureus strain Sau36 chromosome	295	295	100%	1e-77	100.00%	2936968	CP141481
S. aureus strain Sau38 chromosome	295	295	100%	1e-77	100.00%	2798910	CP141477
S. aureus strain Sau104 chromosome	295	295	100%	1e-77	100.00%	2949456	CP141428
S. aureus strain GP12 chromosome	295	295	100%	1e-77	100.00%	2754692	CP139866
S. aureus strain 17CS1042 chromosome	295	295	100%	1e-77	100.00%	2827946	CP138360
S. aureus strain SA31-SX chromosome	295	295	100%	1e-77	100.00%	2777936	CP130510
S. aureus strain 21-024 chromosome	295	295	100%	1e-77	100.00%	2793534	CP128389
S. aureus strain C148 chromosome	295	295	100%	1e-77	100.00%	2858890	CP127743
S. aureus strain C910 chromosome	295	295	100%	1e-77	100.00%	2775416	CP127543
S. aureus strain C878 chromosome	295	295	100%	1e-77	100.00%	2760847	CP127544
S. aureus strain SASWT1215 chromosome	295	295	100%	1e-77	100.00%	2891364	CP109933
S. aureus strain Gv88 chromosome	295	295	100%	1e-77	100.00%	2988028	CP012018
S. aureus strain Be62 chromosome	295	295	100%	1e-77	100.00%	2998480	CP012013
S. aureus strain HC1335 chromosome	295	295	100%	1e-77	100.00%	2976370	CP012012
S. aureus strain 2 chromosome	295	295	100%	1e-77	100.00%	2864598	CP102575
S. aureus strain 1 chromosome	295	295	100%	1e-77	100.00%	2864598	CP102576

Description	Max Score	Total Score	Query Cover	E. value	Per. Ident	Acc. Len	Accession
S. aureus strain 3 chromosome	295	295	100%	1e-77	100.00%	2865930	CP102574
S. aureus strain 12 chromosome	295	295	100%	1e-77	100.00%	2751943	CP102560
S. aureus strain Akali chromosome	295	295	100%	1e-77	100.00%	3022586	CP113032
S. aureus strain Taliyah chromosome	295	295	100%	1e-77	100.00%	3043269	CP113018
S. aureus strain Syndra chromosome	295	295	100%	1e-77	100.00%	2921835	CP113027
S. aureus strain Zed chromosome	295	295	100%	1e-77	100.00%	3048932	CP113015
S. aureus strain Ryze chromosome	295	295	100%	1e-77	100.00%	3019690	CP113007
S. aureus strain VMRSA-WC123 chromosome	295	295	100%	1e-77	100.00%	2898241	CP092538
S. aureus strain VMRSA-WC121 chromosome	295	295	100%	1e-77	100.00%	2898679	CP092540
S. aureus strain VRMSSA-WC113 chromosome	295	295	100%	1e-77	100.00%	2871977	CP092542
S. aureus strain VRMSSA-WC111 chromosome	295	295	100%	1e-77	100.00%	2872486	CP092581
S. aureus strain VMRSA-WC102 chromosome	295	295	100%	1e-77	100.00%	2818951	CP092544
S. aureus strain VMRSA-WC083 chromosome	295	295	100%	1e-77	100.00%	2820042	CP092547
S. aureus strain VMRSA-WC082 chromosome	295	295	100%	1e-77	100.00%	2899492	CP092550
S. aureus strain VMRSA-WC081 chromosome	295	295	100%	1e-77	100.00%	2895422	CP092552
S. aureus strain VMRSA-WC071 chromosome	295	295	100%	1e-77	100.00%	2897962	CP092554
S. aureus strain VMRSA-WC062 chromosome	295	295	100%	1e-77	100.00%	2898133	CP092556
S. aureus strain VMRSA-WC052 chromosome	295	295	100%	1e-77	100.00%	2815072	CP092558
S. aureus strain MRSA-WC101 chromosome	295	295	100%	1e-77	100.00%	2818121	CP092561
S. aureus strain MRSA-WC090 chromosome	295	295	100%	1e-77	100.00%	2820129	CP092563
S. aureus strain MRSA-WC061 chromosome	295	295	100%	1e-77	100.00%	2819747	CP092565
S. aureus strain MRSA-WC000 chromosome	295	295	100%	1e-77	100.00%	2860420	CP092567
S. aureus subsp. aureus SA268	295	295	100%	1e-77	100.00%	2833899	CP006630
S. aureus strain E1185_IV_ST12 chromosome	295	295	100%	1e-77	100.00%	2782853	CP089586
S. aureus strain UNC_SaCF36 chromosome	295	295	100%	1e-77	100.00%	2737790	CP089154
S. aureus strain pt153 chromosome	295	295	100%	1e-77	100.00%	2857481	CP083744
S. aureus isolate HL20835 chromosome	295	295	100%	1e-77	100.00%	2913324	CP080566
S. aureus strain BSN14S3 chromosome	295	295	100%	1e-77	100.00%	2944342	CP080055
S. aureus strain BSN14RB chromosome	295	295	100%	1e-77	100.00%	2944325	CP080054
S. aureus strain BSN14R1 chromosome	295	295	100%	1e-77	100.00%	2944341	CP080053
S. aureus strain PNID0137 chromosome	295	295	100%	1e-77	100.00%	2914236	CP071594
S. aureus strain SA G5 chromosome	295	295	100%	1e-77	100.00%	2760385	CP032160
S. aureus strain pt217 chromosome	295	295	100%	1e-77	100.00%	2907976	CP049510
S. aureus strain pt198 chromosome	295	295	100%	1e-77	100.00%	2912826	CP054269
S. aureus strain ER04648.3 chromosome	295	295	100%	1e-77	100.00%	2900707	CP052028
S. aureus strain ER05322.3 chromosome	295	295	100%	1e-77	100.00%	2872887	CP052025
S. aureus strain ER09403.3 chromosome	295	295	100%	1e-77	100.00%	3005577	CP051976
S. aureus strain ER09761.3 chromosome	295	295	100%	1e-77	100.00%	2895063	CP051958
S. aureus strain ER10234.3 chromosome	295	295	100%	1e-77	100.00%	2867130	CP051939
S. aureus strain ER10920.3 chromosome	295	295	100%	1e-77	100.00%	2871521	CP051917
S. aureus strain ER11063.3 chromosome	295	295	100%	1e-77	100.00%	2969980	CP051914
S. aureus strain ER11789.3 chromosome	295	295	100%	1e-77	100.00%	2855133	CP051895
S. aureus strain pt214 chromosome	295	295	100%	1e-77	100.00%	2832792	CP049994
S. aureus strain pt232 chromosome	295	295	100%	1e-77	100.00%	2938322	CP049991
S. aureus strain pt173 chromosome	295	295	100%	1e-77	100.00%	2907451	CP049580
S. aureus strain pt195 chromosome	295	295	100%	1e-77	100.00%	2898458	CP049545
S. aureus strain pt220 chromosome	295	295	100%	1e-77	100.00%	2903599	CP049503
S. aureus strain pt229 chromosome	295	295	100%	1e-77	100.00%	2937714	CP049485

Description	Max Score	Total Score	Query Cover	E. value	Per. Ident	Acc. Len	Accession
S. aureus strain pt230 chromosome	295	295	100%	1e-77	100.00%	2870752	CP049482
S. aureus strain pt239 chromosome	295	295	100%	1e-77	100.00%	2833595	CP049467
S. aureus strain pt244 chromosome	295	295	100%	1e-77	100.00%	2871690	CP049460
S. aureus strain pt245 chromosome	295	295	100%	1e-77	100.00%	2873667	CP049458
S. aureus strain pt258 chromosome	295	295	100%	1e-77	100.00%	2924234	CP049431
S. aureus strain pt282 chromosome	295	295	100%	1e-77	100.00%	2889597	CP049395
S. aureus strain R24 chromosome	295	295	100%	1e-77	100.00%	2807011	CP060626
S. aureus strain R26 chromosome	295	295	100%	1e-77	100.00%	2807085	CP060625
S. aureus strain R34 chromosome	295	295	100%	1e-77	100.00%	2822220	CP060621
S. aureus strain Sau114 chromosome	290	290	100%	4e-76	99.39%	2924676	CP141419
S. aureus strain JK3137 chromosome	287	287	100%	5e-75	99.39%	2955000	CP020960
S. aureus strain Dresden-275757 chromosome	286	286	100%	5e-75	98.77%	2789663	CP054876
S. aureus strain WCUH29 chromosome	286	286	100%	5e-75	98.77%	2909934	CP039156
S. aureus strain 2288 chromosome	286	286	100%	5e-75	98.77%	2814563	CP026646
S. aureus isolate 20_LA_415, chromosome: I	286	286	100%	5e-75	98.77%	2904888	LT992475
S. aureus isol. 8_LA_272, chromosome: I	286	286	100%	5e-75	98.77%	2891536	LT992461
S. aureus isol. 7_4623, chromosome: I	286	286	100%	5e-75	98.77%	2879411	LT992458
S. aureus strain FDAARGOS_43 chromosome	286	286	100%	5e-75	98.77%	2820631	CP026957
S. aureus strain HPV107 chromosome	286	286	100%	5e-75	98.77%	2877716	CP026074
S. aureus strain Mw2 chromosome	286	286	100%	5e-75	98.77%	2819334	CP026073
S. aureus strain USA400-0051 chromosome	286	286	100%	5e-75	98.77%	2811886	CP019574
S. aureus str. ISU926 isol. ST398 chromosome	286	286	100%	5e-75	98.77%	2833430	CP017091
S. aureus strain LA-MRSA ST398	286	286	100%	5e-75	98.77%	2831848	CP013218
S. aureus strain C96 chromosome	286	286	100%	5e-75	98.77%	2793863	CP127773
S. aureus strain C133 chromosome	286	286	100%	5e-75	98.77%	2800426	CP127702
S. aureus strain C179 chromosome	286	286	100%	5e-75	98.77%	2800426	CP127700
S. aureus subsp. aureus strain GR2	286	286	100%	5e-75	98.77%	2792802	CP010402
S. aureus NCTC13435, chromosome	286	286	100%	5e-75	98.77%	2797452	LN831036
S. aureus strain IVB6165 chromosome	286	286	100%	5e-75	98.77%	2785555	CP094793
S. aureus strain IVB6173 chromosome	286	286	100%	5e-75	98.77%	2785549	CP094780
S. aureus strain IVB6221 chromosome	286	286	100%	5e-75	98.77%	2809177	CP094763
S. aureus strain IVB6248 chromosome	286	286	100%	5e-75	98.77%	2786100	CP094750
S. aureus strain IVB6252 chromosome	286	286	100%	5e-75	98.77%	2785956	CP094743
S. aureus strain N12HSA28 chromosome	286	286	100%	5e-75	98.77%	2833771	CP091523
S. aureus strain 55-100-016 chromosome	286	286	100%	5e-75	98.77%	2809746	CP076839
S. aureus strain M20 chromosome	286	286	100%	5e-75	98.77%	2806436	CP071348
S. aureus strain M37 chromosome	286	286	100%	5e-75	98.77%	2798511	CP071347
S. aureus subsp. aureus 11819-97	286	286	100%	5e-75	98.77%	2846546	CP003194
S. aureus strain 111250134 chromosome	286	286	100%	5e-75	98.77%	2897518	CP045442
S. aureus strain 950122 chromosome	286	286	100%	5e-75	98.77%	2809177	CP045441
S. aureus subsp. aureus MW2 DNA	286	286	100%	5e-75	98.77%	2820462	BA000033
S. aureus strain Ahri chromosome	282	282	100%	6e-74	98.79%	2923858	CP113049

*The search was performed with the use of BlastN, using the complete genomic sequences deposited in GenBank (accessed 2024-10-23) and the 163 bp region between the *immR* and *cro* gene of ASZ22RN as a query (see Fig. S4).

Table S9. *Staphylococcus aureus* strains encoding prophage ImmR proteins identical or nearly identical to that of ASZ22RN

Description	Max Score	Total Score	Query Cover	E. value	Per. Ident	Acc. Len	Accession
S. aureus strain CC479-MSSA chromosome	219	219	100%	1e-65	100.00%	2736525	CP155061
S. aureus strain 7-7 chromosome	219	219	100%	1e-65	100.00%	2737750	CP126626
S. aureus strain UNC_SaCF36 chromosome	219	219	100%	1e-65	100.00%	2737790	CP089154
S. aureus strain CC479-MSSA chromosome	219	219	100%	1e-65	100.00%	2739900	CP155058
S. aureus strain 18-22 chromosome	219	219	100%	1e-65	100.00%	2741653	CP126630
S. aureus strain 33-40 chromosome	219	219	100%	1e-65	100.00%	2742607	CP126631
S. aureus strain 35-42 chromosome	219	219	100%	1e-65	100.00%	2742763	CP126629
S. aureus strain 12 chromosome	219	219	100%	1e-65	100.00%	2751943	CP102560
S. aureus strain GP12 chromosome	219	219	100%	1e-65	100.00%	2754692	CP139866
S. aureus strain SA G5 chromosome	219	219	100%	1e-65	100.00%	2760385	CP032160
S. aureus strain C878 chromosome	219	219	100%	1e-65	100.00%	2760847	CP127544
S. aureus DNA strain: No.10	219	219	100%	1e-65	100.00%	2764435	AP015012
S. aureus strain 24EBSta0529 chromosome	219	219	100%	1e-65	100.00%	2767774	CP166872
S. aureus strain 30366_IOW744N4 chromosome	219	219	100%	1e-65	100.00%	2775300	CP069351
S. aureus strain C910 chromosome	219	219	100%	1e-65	100.00%	2775416	CP127543
S. aureus strain RIVM1295 chromosome	219	219	100%	1e-65	100.00%	2777077	CP013616
S. aureus strain SA31-SX chromosome	219	219	100%	1e-65	100.00%	2777936	CP130510
S. aureus strain IPLA15 chromosome	219	219	100%	1e-65	100.00%	2781582	CP134618
S. aureus strain B4-59C chromosome	219	219	100%	1e-65	100.00%	2781709	CP042153
S. aureus strain E1185_IV_ST12 chromosome	219	219	100%	1e-65	100.00%	2782853	CP089586
S. aureus strain 21-024 chromosome	219	219	100%	1e-65	100.00%	2793534	CP128389
S. aureus strain O55 isol. B118 chromosome	219	219	100%	1e-65	100.00%	2794042	CP038268
S. aureus strain Sau38 chromosome	219	219	100%	1e-65	100.00%	2798910	CP141477
S. aureus strain RIVM_M047065 chromosome	219	219	100%	1e-65	100.00%	2806671	CP096539
S. aureus strain B2-15A chromosome	219	219	100%	1e-65	100.00%	2806828	CP042043
S. aureus strain UP_1632 chromosome	219	329	100%	1e-65	100.00%	2806864	CP047777
S. aureus strain R24 chromosome	219	219	100%	1e-65	100.00%	2807011	CP060626
S. aureus strain R26 chromosome	219	219	100%	1e-65	100.00%	2807085	CP060625
S. aureus strain B8-13D chromosome	219	219	100%	1e-65	100.00%	2807514	CP042107
S. aureus strain IVB6196 chromosome	219	219	100%	1e-65	100.00%	2808124	CP094772
S. aureus strain BSN142 chromosome	219	219	100%	1e-65	100.00%	2808584	CP151289
S. aureus strain IVB6243 chromosome	219	219	100%	1e-65	100.00%	2813486	CP094752
S. aureus strain IVB6242 chromosome	219	219	100%	1e-65	100.00%	2813489	CP094755
S. aureus strain BSN180 chromosome	219	219	100%	1e-65	100.00%	2813540	CP150881
S. aureus strain VMRSA-WC052 chromosome	219	219	100%	1e-65	100.00%	2815072	CP092558
S. aureus strain CUBIST-10 chromosome	219	219	100%	1e-65	100.00%	2816922	CP167764
S. aureus strain MRSA-WC101 chromosome	219	219	100%	1e-65	100.00%	2818121	CP092561
S. aureus strain VMRSA-WC102 chromosome	219	219	100%	1e-65	100.00%	2818951	CP092544
S. aureus strain MRSA-WC061 chromosome	219	219	100%	1e-65	100.00%	2819747	CP092565
S. aureus strain VMRSA-WC083 chromosome	219	219	100%	1e-65	100.00%	2820042	CP092547
S. aureus strain MRSA-WC090 chromosome	219	219	100%	1e-65	100.00%	2820129	CP092563
S. aureus strain R34 chromosome	219	219	100%	1e-65	100.00%	2822220	CP060621
S. aureus strain ch21 chromosome	219	219	100%	1e-65	100.00%	2822599	CP017804
S. aureus subsp. aureus ED98	219	219	100%	1e-65	100.00%	2824404	CP001781
S. aureus strain 17CS1042 chromosome	219	219	100%	1e-65	100.00%	2827946	CP138360

S. aureus strain ER02988.3 chromosome	219	219	100%	1e-65	100.00%	2828341	CP030588
S. aureus strain pt214 chromosome	219	219	100%	1e-65	100.00%	2832792	CP049994
S. aureus strain pt239 chromosome	219	219	100%	1e-65	100.00%	2833595	CP049467
S. aureus subsp. aureus SA268	219	219	100%	1e-65	100.00%	2833899	CP006630
S. aureus strain UCI 28 isolate ST5 chromosome	219	219	100%	1e-65	100.00%	2835307	CP018768
S. aureus strain ER04164.3 chromosome	219	219	100%	1e-65	100.00%	2837559	CP030542
S. aureus strain UP_551 chromosome	219	219	100%	1e-65	100.00%	2840057	CP047794
S. aureus strain B3-17D chromosome	219	219	100%	1e-65	100.00%	2840146	CP042157
S. aureus strain Guangzhou-SAU749 chromosome	219	219	100%	1e-65	100.00%	2840643	CP053185
S. aureus strain BSN85 chromosome	219	219	100%	1e-65	100.00%	2842199	CP157306
S. aureus CA-347	219	219	100%	1e-65	100.00%	2850503	CP006044
S. aureus strain ER01009.3 chromosome	219	219	100%	1e-65	100.00%	2852702	CP030489
S. aureus strain ER04402.3 chromosome	219	219	100%	1e-65	100.00%	2853044	CP030505
S. aureus strain IVB6156 chromosome	219	219	100%	1e-65	100.00%	2853773	CP095116
S. aureus strain ER11789.3 chromosome	219	219	100%	1e-65	100.00%	2855133	CP051895
S. aureus strain N29CSA02 chromosome	219	219	100%	1e-65	100.00%	2855262	CP119341
S. aureus strain BSN25 chromosome	219	219	100%	1e-65	100.00%	2856082	CP158667
S. aureus strain 2010N06-097 chromosome	219	219	100%	1e-65	100.00%	2857030	CP131655
S. aureus strain CFSAN007847 chromosome	219	219	100%	1e-65	100.00%	2857060	CP017684
S. aureus strain pt153 chromosome	219	219	100%	1e-65	100.00%	2857481	CP083744
S. aureus strain ER03737.3 chromosome	219	329	100%	1e-65	100.00%	2857621	CP030641
S. aureus strain C148 chromosome	219	219	100%	1e-65	100.00%	2858890	CP127743
S. aureus strain MRSA-WC000 chromosome	219	219	100%	1e-65	100.00%	2860420	CP092567
S. aureus strain ER02919.3 chromosome	219	219	100%	1e-65	100.00%	2861613	CP030688
S. aureus strain BSN129 chromosome	219	341	100%	1e-65	100.00%	2862598	CP151822
S. aureus strain 1 chromosome	219	219	100%	1e-65	100.00%	2864598	CP102576
S. aureus strain 2 chromosome	219	219	100%	1e-65	100.00%	2864598	CP102575
S. aureus strain ER04567.3 chromosome	219	219	100%	1e-65	100.00%	2865586	CP030411
S. aureus strain 3 chromosome	219	219	100%	1e-65	100.00%	2865930	CP102574
S. aureus strain ER10234.3 chromosome	219	219	100%	1e-65	100.00%	2867130	CP051939
S. aureus strain ER01935.3 chromosome	219	219	100%	1e-65	100.00%	2870160	CP030540
S. aureus strain pt230 chromosome	219	219	100%	1e-65	100.00%	2870752	CP049482
S. aureus strain ER10920.3 chromosome	219	219	100%	1e-65	100.00%	2871521	CP051917
S. aureus strain pt244 chromosome	219	219	100%	1e-65	100.00%	2871690	CP049460
S. aureus strain ER10667.3 chromosome	219	219	100%	1e-65	100.00%	2871890	CP051929
S. aureus strain VRMSSA-WC113 chromosome	219	219	100%	1e-65	100.00%	2871977	CP092542
S. aureus strain VRMSSA-WC111 chromosome	219	219	100%	1e-65	100.00%	2872486	CP092581
S. aureus strain ER05322.3 chromosome	219	219	100%	1e-65	100.00%	2872887	CP052025
S. aureus strain ER03864.3 chromosome	219	219	100%	1e-65	100.00%	2873608	CP030566
S. aureus strain pt245 chromosome	219	219	100%	1e-65	100.00%	2873667	CP049458
S. aureus strain CNRS22817 chromosome	219	219	100%	1e-65	100.00%	2874629	CP140690
S. aureus isolate 22_LA_562 ass. chromosome	219	219	100%	1e-65	100.00%	2875548	LT992477
S. aureus strain ER01532.3 chromosome	219	219	100%	1e-65	100.00%	2876387	CP030695
S. aureus strain ER04174.3 chromosome	219	219	100%	1e-65	100.00%	2877642	CP030525
S. aureus strain SA01 chromosome	219	219	100%	1e-65	100.00%	2883539	CP053075
S. aureus strain 2868B2 chromosome	219	219	100%	1e-65	100.00%	2884711	CP060141
S. aureus strain pt246 chromosome	219	219	100%	1e-65	100.00%	2888623	CP049454
S. aureus strain pt282 chromosome	219	219	100%	1e-65	100.00%	2889597	CP049395
S. aureus strain SASWT1215 chromosome	219	219	100%	1e-65	100.00%	2891364	CP109933
S. aureus strain ER10115.3 chromosome	219	219	100%	1e-65	100.00%	2892920	CP051945

S. aureus strain FDAARGOS_504 chromosome	219	219	100%	1e-65	100.00%	2894587	CP033865
S. aureus strain ER09761.3 chromosome	219	219	100%	1e-65	100.00%	2895063	CP051958
S. aureus strain VMRSA-WC081 chromosome	219	219	100%	1e-65	100.00%	2895422	CP092552
S. aureus strain VMRSA-WC071 chromosome	219	219	100%	1e-65	100.00%	2897962	CP092554
S. aureus strain VMRSA-WC062 chromosome	219	219	100%	1e-65	100.00%	2898133	CP092556
S. aureus strain VMRSA-WC123 chromosome	219	219	100%	1e-65	100.00%	2898241	CP092538
S. aureus strain pt195 chromosome	219	219	100%	1e-65	100.00%	2898458	CP049545
S. aureus strain VMRSA-WC121 chromosome	219	219	100%	1e-65	100.00%	2898679	CP092540
S. aureus strain VMRSA-WC082 chromosome	219	219	100%	1e-65	100.00%	2899492	CP092550
S. aureus strain ER00551.3 chromosome	219	219	100%	1e-65	100.00%	2900663	CP030424
S. aureus strain ER04648.3 chromosome	219	219	100%	1e-65	100.00%	2900707	CP052028
S. aureus strain ER11327.3 chromosome	219	219	100%	1e-65	100.00%	2900855	CP051909
S. aureus strain BSN71 chromosome	219	219	100%	1e-65	100.00%	2901695	CP157302
S. aureus strain pt220 chromosome	219	219	100%	1e-65	100.00%	2903599	CP049503
S. aureus strain AR_474 chromosome	219	329	100%	1e-65	100.00%	2906506	CP030326
S. aureus subsp. aureus JH9	219	329	100%	1e-65	100.00%	2906700	CP000703
S. aureus strain ER04181.3 chromosome	219	219	100%	1e-65	100.00%	2907452	CP030547
S. aureus strain pt173 chromosome	219	219	100%	1e-65	100.00%	2907451	CP049580
S. aureus strain pt217 chromosome	219	219	100%	1e-65	100.00%	2907976	CP049510
S. aureus strain ER01422.3 chromosome	219	219	100%	1e-65	100.00%	2908097	CP030692
S. aureus strain ER04219.3 chromosome	219	219	100%	1e-65	100.00%	2908316	CP030404
S. aureus strain ER03444.3 chromosome	219	219	100%	1e-65	100.00%	2911432	CP030475
S. aureus strain 191 chromosome	219	219	100%	1e-65	100.00%	2911744	CP022894
S. aureus strain 61 chromosome	219	219	100%	1e-65	100.00%	2911744	CP022893
S. aureus strain 628 chromosome	219	219	100%	1e-65	100.00%	2911773	CP022905
S. aureus strain BSN66 chromosome	219	219	100%	1e-65	100.00%	2912240	CP150828
S. aureus strain pt198 chromosome	219	219	100%	1e-65	100.00%	2912826	CP054269
S. aureus strain 187 chromosome	219	219	100%	1e-65	100.00%	2913163	CP022903
S. aureus strain pt192 chromosome	219	219	100%	1e-65	100.00%	2913317	CP049551
S. aureus isolate HL20835 chromosome	219	219	100%	1e-65	100.00%	2913324	CP080566
S. aureus strain 629 chromosome	219	219	100%	1e-65	100.00%	2913334	CP022904
S. aureus strain PNID0137 chromosome	219	219	100%	1e-65	100.00%	2914236	CP071594
S. aureus strain 16439 chromosome	219	219	100%	1e-65	100.00%	2915632	CP043300
S. aureus strain ER09706.3 chromosome	219	219	100%	1e-65	100.00%	2916678	CP051961
S. aureus strain CUBIST-25 chromosome	219	219	100%	1e-65	100.00%	2921731	CP168087
S. aureus strain ER00959.3 chromosome	219	219	100%	1e-65	100.00%	2921825	CP030384
S. aureus strain Syndra chromosome	219	219	100%	1e-65	100.00%	2921835	CP113027
S. aureus isolate 17_LA_343 ass. chromosome	219	219	100%	1e-65	100.00%	2922778	LT992471
S. aureus strain pt258 chromosome	219	219	100%	1e-65	100.00%	2924234	CP049431
S. aureus strain Sau26 chromosome	219	219	100%	1e-65	100.00%	2925858	CP141490
S. aureus strain BSN188 chromosome	219	219	100%	1e-65	100.00%	2927162	CP156766
S. aureus strain BSN49 chromosome	219	341	100%	1e-65	100.00%	2931131	CP159794
S. aureus strain E1 chromosome	219	219	100%	1e-65	100.00%	2931383	CP157976
S. aureus strain pt296 chromosome	219	219	100%	1e-65	100.00%	2932301	CP049379
S. aureus strain BSN48-2 chromosome	219	341	100%	1e-65	100.00%	2932403	CP157420
S. aureus strain 545 chromosome	219	219	100%	1e-65	100.00%	2935463	CP022908
S. aureus strain ER02360.3 chromosome	219	219	100%	1e-65	100.00%	2936067	CP030393
S. aureus strain A1 chromosome	219	219	100%	1e-65	100.00%	2936542	CP140612
S. aureus strain ER00503.3 chromosome	219	219	100%	1e-65	100.00%	2936779	CP030493
S. aureus strain Sau36 chromosome	219	219	100%	1e-65	100.00%	2936968	CP141481

S. aureus strain pt229 chromosome	219	219	100%	1e-65	100.00%	2937714	CP049485
S. aureus strain pt232 chromosome	219	219	100%	1e-65	100.00%	2938322	CP049991
S. aureus strain NV_1 chromosome	219	219	100%	1e-65	100.00%	2939569	CP080249
S. aureus strain 16445 chromosome	219	327	100%	1e-65	100.00%	2940576	CP043302
S. aureus strain BSN14RB chromosome	219	219	100%	1e-65	100.00%	2944325	CP080054
S. aureus strain BSN14R1 chromosome	219	219	100%	1e-65	100.00%	2944341	CP080053
S. aureus strain BSN14S3 chromosome	219	219	100%	1e-65	100.00%	2944342	CP080055
S. aureus strain E2 chromosome	219	219	100%	1e-65	100.00%	2944378	CP157977
S. aureus strain BSN100 chromosome	219	219	100%	1e-65	100.00%	2944470	CP149463
S. aureus strain E3 chromosome	219	219	100%	1e-65	100.00%	2944807	CP157978
S. aureus strain Sau104 chromosome	219	219	100%	1e-65	100.00%	2949456	CP141428
S. aureus strain UP_1452 chromosome	219	329	100%	1e-65	100.00%	2949972	CP047781
S. aureus strain pt293 chromosome	219	219	100%	1e-65	100.00%	2950069	CP049381
S. aureus strain BSN207 chromosome	219	219	100%	1e-65	100.00%	2954066	CP158422
S. aureus strain JK3137 chromosome	219	219	100%	1e-65	100.00%	2955000	CP020960
S. aureus strain 2395 USA500	219	219	100%	1e-65	100.00%	2955646	CP007499
S. aureus strain TUM22702 chromosome	219	341	100%	1e-65	100.00%	2955722	CP150744
S. aureus strain TUM22702 chromosome	219	341	100%	1e-65	100.00%	2955727	CP162430
S. aureus strain NRS120 chromosome	219	219	100%	1e-65	100.00%	2955890	CP026072
S. aureus strain TUM22701 chromosome	219	341	100%	1e-65	100.00%	2958459	CP150747
S. aureus strain TUM22701 chromosome	219	341	100%	1e-65	100.00%	2959317	CP162433
S. aureus strain CUBIST-16 chromosome	219	219	100%	1e-65	100.00%	2961621	CP168091
S. aureus strain BSN82 chromosome	219	219	100%	1e-65	100.00%	2964297	CP157308
S. aureus strain ER02836.3 chromosome	219	219	100%	1e-65	100.00%	2965519	CP030432
S. aureus strain BSN94 chromosome	219	219	100%	1e-65	100.00%	2967725	CP151423
S. aureus strain ER02878.3 chromosome	219	219	100%	1e-65	100.00%	2968616	CP030713
S. aureus strain ER11063.3 chromosome	219	219	100%	1e-65	100.00%	2969980	CP051914
S. aureus strain HC1335 chromosome	219	329	100%	1e-65	100.00%	2976370	CP012012
S. aureus strain Gv88 chromosome	219	329	100%	1e-65	100.00%	2988028	CP012018
S. aureus strain Be62 chromosome	219	308	100%	1e-65	100.00%	2998480	CP012013
S. aureus strain ER09403.3 chromosome	219	219	100%	1e-65	100.00%	3005577	CP051976
S. aureus strain Ryze chromosome	219	219	100%	1e-65	100.00%	3019690	CP113007
S. aureus strain Akali chromosome	219	219	100%	1e-65	100.00%	3022586	CP113032
S. aureus strain Taliyah chromosome	219	219	100%	1e-65	100.00%	3043269	CP113018
S. aureus strain Zed chromosome	219	219	100%	1e-65	100.00%	3048932	CP113015
S. aureus strain ch22 chromosome	219	219	100%	1e-65	100.00%	3074078	CP017807
S. aureus strain MRSA107 chromosome	219	219	100%	1e-65	100.00%	3095697	CP018629
S. aureus strain nan_175_F371_ch chromosome	219	219	100%	3e-65	99.07%	2793849	CP066492
S. aureus strain ncr_155_F133 chromosome	219	264	100%	3e-65	99.07%	2820908	CP066488
S. aureus isolate 6_LA_232 ass. chromosome	218	218	100%	6e-65	99.07%	2843512	LT992465
S. aureus strain IVB6170 chromosome	217	217	100%	1e-64	99.07%	2766604	CP094783
S. aureus strain IVB6239 chromosome	217	217	100%	1e-64	99.07%	2766759	CP094758
S. aureus ass.	217	217	100%	1e-64	99.07%	2870460	CP118810
S._aureus_ILRI_Eymole1/1 chromosome	217	217	100%	1e-64	99.07%	2874302	LN626917
S. aureus strain Sau86 chromosome	217	217	100%	1e-64	99.07%	2895676	CP141440
S. aureus strain Sau6 chromosome	217	217	100%	1e-64	99.07%	2900088	CP141512
S. aureus strain RGB-095930 chromosome	217	217	100%	1e-64	99.07%	2904556	CP077098
S. aureus strain IVB6169 chromosome	217	217	100%	1e-64	99.07%	2917187	CP094785
S. aureus strain IVB6167 chromosome	217	217	100%	1e-64	99.07%	2917188	CP094789
S. aureus strain IVB6166 chromosome	217	217	100%	1e-64	99.07%	2917190	CP094791

S. aureus strain IVB6173 chromosome	217	217	100%	1e-64	99.07%	2785549	CP094780
S. aureus strain IVB6165 chromosome	217	217	100%	1e-64	99.07%	2785555	CP094793
S. aureus strain IVB6252 chromosome	217	217	100%	1e-64	99.07%	2785956	CP094743
S. aureus strain IVB6248 chromosome	217	217	100%	1e-64	99.07%	2786100	CP094750
S. aureus strain Dresden-275757 chromosome	217	217	100%	1e-64	99.07%	2789663	CP054876
S. aureus strain GD705 chromosome	217	217	100%	1e-64	99.07%	2790096	CP019593
S. aureus subsp. aureus strain GR2	217	217	100%	1e-64	99.07%	2792802	CP010402
S. aureus ass. NCTC13435 chromosome	217	217	100%	1e-64	99.07%	2797452	LN831036
S. aureus strain IVB6221 chromosome	217	217	100%	1e-64	99.07%	2809177	CP094763
S. aureus strain N12HSA28 chromosome	217	217	100%	1e-64	99.07%	2833771	CP091523
S. aureus subsp. aureus 11819-97	217	217	100%	1e-64	99.07%	2846546	CP003194
S. aureus strain HPV107 chromosome	217	217	100%	1e-64	99.07%	2877716	CP026074
S. aureus strain WCUH29 chromosome	217	217	100%	1e-64	99.07%	2909934	CP039156
S. aureus strain 18 chromosome	215	325	100%	6e-64	98.13%	2870995	CP094924
S. aureus strain MW2-16 chromosome	215	215	100%	7e-64	98.13%	2777737	CP151929
S. aureus strain C96 chromosome	215	215	100%	7e-64	98.13%	2793863	CP127773
S. aureus strain M37 chromosome	215	215	100%	7e-64	98.13%	2798511	CP071347
S. aureus strain C133 chromosome	215	215	100%	7e-64	98.13%	2800426	CP127702
S. aureus strain C179 chromosome	215	215	100%	7e-64	98.13%	2800426	CP127700
S. aureus strain M20 chromosome	215	215	100%	7e-64	98.13%	2806436	CP071348
S. aureus strain 950122 chromosome	215	215	100%	7e-64	98.13%	2809177	CP045441
S. aureus strain 55-100-016 chromosome	215	215	100%	7e-64	98.13%	2809746	CP076839
S. aureus strain USA400-0051 chromosome	215	215	100%	7e-64	98.13%	2811886	CP019574
S. aureus strain 2288 chromosome	215	215	100%	7e-64	98.13%	2814563	CP026646
S. aureus strain Mw2 chromosome	215	215	100%	7e-64	98.13%	2819334	CP026073
S. aureus strain MW2-47 chromosome	215	215	100%	7e-64	98.13%	2820174	CP151922
S. aureus strain MW2-S1 chromosome	215	215	100%	7e-64	98.13%	2820462	CP151930
S. aureus subsp. aureus MW2 DNA	215	215	100%	7e-64	98.13%	2820462	BA000033
S. aureus strain MW2-45 chromosome	215	215	100%	7e-64	98.13%	2820473	CP151923
S. aureus strain MW2-48 chromosome	215	215	100%	7e-64	98.13%	2820507	CP151921
S. aureus strain MW2-25 chromosome	215	215	100%	7e-64	98.13%	2820516	CP151927
S. aureus strain MW2-35 chromosome	215	215	100%	7e-64	98.13%	2820538	CP151925
S. aureus strain MW2-23 chromosome	215	215	100%	7e-64	98.13%	2820541	CP151928
S. aureus strain MW2-32 chromosome	215	215	100%	7e-64	98.13%	2820554	CP151926
S. aureus strain MW2-42 chromosome	215	215	100%	7e-64	98.13%	2820590	CP151924
S. aureus strain FDAARGOS_43 chromosome	215	215	100%	7e-64	98.13%	2820631	CP026957
S. aureus strain MW2-50 chromosome	215	215	100%	7e-64	98.13%	2820698	CP151920
S. aureus strain NZ15MR0322 ass. chromosome:	215	215	100%	7e-64	98.13%	2839203	LT699704
S. aureus strain 14 chromosome	215	325	100%	7e-64	98.13%	2856550	CP094926
S. aureus strain 15 chromosome	215	325	100%	7e-64	98.13%	2856558	CP094925
S. aureus strain 1824 chromosome	215	325	100%	7e-64	98.13%	2871168	CP094923
S. aureus strain 13 chromosome	215	325	100%	7e-64	98.13%	2872257	CP094927
S. aureus isolate 7_4623 ass. chromosome:	215	215	100%	7e-64	98.13%	2879411	LT992458
S. aureus isolate 8_LA_272 ass. chromosome:	215	215	100%	7e-64	98.13%	2891536	LT992461
S. aureus strain 111250134 chromosome	215	215	100%	7e-64	98.13%	2897518	CP045442
S. aureus isolate 20_LA_415 ass. chromosome:	215	215	100%	7e-64	98.13%	2904888	LT992475
S. aureus isolate E4_22S00627-1 ass. chromosome	215	215	100%	7e-64	98.13%	2922701	OZ059818
S. aureus strain Sau114 chromosome	215	215	100%	7e-64	98.13%	2924676	CP141419
S. aureus isolate E1_22S00622-1 ass. chromosome	215	215	100%	7e-64	98.13%	2927810	OZ059820
S. aureus isolate E2_22S00623-1 ass. chromosome	215	215	100%	7e-64	98.13%	2927838	OZ059823

S. aureus isolate H2_22S00625-1 ass. chromosome	215	215	100%	7e-64	98.13%	2927893	OZ059816
S. aureus isolate E3_22S00626-1 ass. chromosome	215	215	100%	7e-64	98.13%	2927898	OZ059824

*The search was performed with the use of tBlastN, using the complete genomic sequences deposited in GenBank (accessed 2024-10-23) and the ASZ22RN ImmR protein sequence as a query.

Table S10. *Staphylococcus aureus* strains encoding prophage Cro proteins identical or nearly identical to that of ASZ22RN

Description	Max Score	Total Score	Query Cover	E. value	Per. Ident	Acc. Len	Accession
S. aureus strain UNC_SaCF36 chromosome	171	171	100%	4e-49	100.00%	2737790	CP089154
S. aureus strain 12 chromosome	171	171	100%	4e-49	100.00%	2751943	CP102560
S. aureus strain SA G5 chromosome	171	171	100%	4e-49	100.00%	2760385	CP032160
S. aureus DNA, almost complete, strain: No.10	171	171	100%	4e-49	100.00%	2764435	AP015012
S. aureus strain IVB6170 chromosome	171	171	100%	4e-49	100.00%	2766604	CP094783
S. aureus strain IVB6239 chromosome	171	171	100%	4e-49	100.00%	2766759	CP094758
S. aureus strain RIVM1295 chromosome	171	171	100%	4e-49	100.00%	2777077	CP013616
S. aureus strain SA31-SX chromosome	171	171	100%	4e-49	100.00%	2777936	CP130510
S. aureus strain IPLA15 chromosome	171	171	100%	4e-49	100.00%	2781582	CP134618
S. aureus strain E1185_IV_ST12 chromosome	171	171	100%	4e-49	100.00%	2782853	CP089586
S. aureus strain 21-024 chromosome	171	171	100%	4e-49	100.00%	2793534	CP128389
S. aureus strain UP_1632 chromosome	171	171	100%	4e-49	100.00%	2806864	CP047777
S. aureus strain R24 chromosome	171	171	100%	4e-49	100.00%	2807011	CP060626
S. aureus strain R26 chromosome	171	171	100%	4e-49	100.00%	2807085	CP060625
S. aureus strain VMRSA-WC052 chromosome	171	171	100%	4e-49	100.00%	2815072	CP092558
S. aureus strain MRSA-WC101 chromosome	171	171	100%	4e-49	100.00%	2818121	CP092561
S. aureus strain VMRSA-WC102 chromosome	171	171	100%	4e-49	100.00%	2818951	CP092544
S. aureus strain MRSA-WC061 chromosome	171	171	100%	4e-49	100.00%	2819747	CP092565
S. aureus strain VMRSA-WC083 chromosome	171	171	100%	4e-49	100.00%	2820042	CP092547
S. aureus strain MRSA-WC090 chromosome	171	171	100%	4e-49	100.00%	2820129	CP092563
S. aureus strain R34 chromosome	171	171	100%	4e-49	100.00%	2822220	CP060621
S. aureus strain ER02988.3 chromosome	171	171	100%	4e-49	100.00%	2828341	CP030588
S. aureus strain pt214 chromosome	171	171	100%	4e-49	100.00%	2832792	CP049994
S. aureus subsp. aureus SA268	171	171	100%	4e-49	100.00%	2833899	CP006630
S. aureus strain UCI 28 ST5 chromosome	171	171	100%	4e-49	100.00%	2835307	CP018768
S. aureus strain UP_551 chromosome	171	171	100%	4e-49	100.00%	2840057	CP047794
S. aureus strain Guangzhou-SAU749 chromosome	171	171	100%	4e-49	100.00%	2840643	CP053185
S. aureus isolate 6_LA_232, chromosome: I	171	171	100%	4e-49	100.00%	2843512	LT992465
S. aureus strain ER01009.3 chromosome	171	171	100%	4e-49	100.00%	2852702	CP030489
S. aureus strain ER11789.3 chromosome	171	171	100%	4e-49	100.00%	2855133	CP051895
S. aureus strain 2010N06-097 chromosome	171	171	100%	4e-49	100.00%	2857030	CP131655
S. aureus strain CFSAN007847 chromosome	171	171	100%	4e-49	100.00%	2857060	CP017684
S. aureus strain pt153 chromosome	171	171	100%	4e-49	100.00%	2857481	CP083744
S. aureus strain C148 chromosome	171	171	100%	4e-49	100.00%	2858890	CP127743
S. aureus strain MRSA-WC000 chromosome	171	171	100%	4e-49	100.00%	2860420	CP092567
S. aureus strain 1 chromosome	171	171	100%	4e-49	100.00%	2864598	CP102576
S. aureus strain 2 chromosome	171	171	100%	4e-49	100.00%	2864598	CP102575

Description	Max Score	Total Score	Query Cover	E. value	Per. Ident	Acc. Len	Accession
S. aureus strain ER04567.3 chromosome	171	171	100%	4e-49	100.00%	2865586	CP030411
S. aureus strain 3 chromosome	171	171	100%	4e-49	100.00%	2865930	CP102574
S. aureus strain ER01935.3 chromosome	171	171	100%	4e-49	100.00%	2870160	CP030540
S. aureus strain pt230 chromosome	171	171	100%	4e-49	100.00%	2870752	CP049482
S. aureus strain ER10920.3 chromosome	171	171	100%	4e-49	100.00%	2871521	CP051917
S. aureus strain pt244 chromosome	171	171	100%	4e-49	100.00%	2871690	CP049460
S. aureus strain VRMSSA-WC113 chromosome	171	171	100%	4e-49	100.00%	2871977	CP092542
S. aureus strain VRMSSA-WC111 chromosome	171	171	100%	4e-49	100.00%	2872486	CP092581
S. aureus strain ER05322.3 chromosome	171	171	100%	4e-49	100.00%	2872887	CP052025
S. aureus strain ER03864.3 chromosome	171	171	100%	4e-49	100.00%	2873608	CP030566
S. aureus strain pt245 chromosome	171	171	100%	4e-49	100.00%	2873667	CP049458
S._aureus_ILRI_Eymole1/1, chromosome : I	171	171	100%	4e-49	100.00%	2874302	LN626917
S. aureus strain CNRS22817 chromosome	171	171	100%	4e-49	100.00%	2874629	CP140690
S. aureus isolate 22_LA_562, chromosome: I	171	171	100%	4e-49	100.00%	2875548	LT992477
S. aureus strain ER04174.3 chromosome	171	171	100%	4e-49	100.00%	2877642	CP030525
S. aureus strain pt282 chromosome	171	171	100%	4e-49	100.00%	2889597	CP049395
S. aureus strain SASWT1215 chromosome	171	171	100%	4e-49	100.00%	2891364	CP109933
S. aureus strain ER10115.3 chromosome	171	171	100%	4e-49	100.00%	2892920	CP051945
S. aureus strain ER09761.3 chromosome	171	171	100%	4e-49	100.00%	2895063	CP051958
S. aureus strain VMRSA-WC081 chromosome	171	171	100%	4e-49	100.00%	2895422	CP092552
S. aureus strain VMRSA-WC071 chromosome	171	171	100%	4e-49	100.00%	2897962	CP092554
S. aureus strain VMRSA-WC062 chromosome	171	171	100%	4e-49	100.00%	2898133	CP092556
S. aureus strain VMRSA-WC123 chromosome	171	171	100%	4e-49	100.00%	2898241	CP092538
S. aureus strain pt195 chromosome	171	171	100%	4e-49	100.00%	2898458	CP049545
S. aureus strain VMRSA-WC121 chromosome	171	171	100%	4e-49	100.00%	2898679	CP092540
S. aureus strain VMRSA-WC082 chromosome	171	171	100%	4e-49	100.00%	2899492	CP092550
S. aureus strain ER01422.3 chromosome	171	171	100%	4e-49	100.00%	2908097	CP030692
S. aureus strain ER04219.3 chromosome	171	171	100%	4e-49	100.00%	2908316	CP030404
S. aureus strain ER03444.3 chromosome	171	171	100%	4e-49	100.00%	2911432	CP030475
S. aureus strain 191 chromosome	171	171	100%	4e-49	100.00%	2911744	CP022894
S. aureus strain 61 chromosome	171	171	100%	4e-49	100.00%	2911744	CP022893
S. aureus strain 628 chromosome	171	171	100%	4e-49	100.00%	2911773	CP022905
S. aureus strain BSN66 chromosome	171	171	100%	4e-49	100.00%	2912240	CP150828
S. aureus strain pt198 chromosome	171	171	100%	4e-49	100.00%	2912826	CP054269
S. aureus strain 187 chromosome	171	171	100%	4e-49	100.00%	2913163	CP022903
S. aureus isolate HL20835 chromosome	171	171	100%	4e-49	100.00%	2913324	CP080566
S. aureus strain 629 chromosome	171	171	100%	4e-49	100.00%	2913334	CP022904
S. aureus strain PNID0137 chromosome	171	171	100%	4e-49	100.00%	2914236	CP071594
S. aureus strain IVB6169 chromosome	171	171	100%	4e-49	100.00%	2917187	CP094785
S. aureus strain IVB6167 chromosome	171	171	100%	4e-49	100.00%	2917188	CP094789
S. aureus strain IVB6166 chromosome	171	171	100%	4e-49	100.00%	2917190	CP094791
S. aureus strain ER00959.3 chromosome	171	171	100%	4e-49	100.00%	2921825	CP030384
S. aureus isolate 17_LA_343, chromosome: I	171	171	100%	4e-49	100.00%	2922778	LT992471
S. aureus strain pt258 chromosome	171	171	100%	4e-49	100.00%	2924234	CP049431
S. aureus strain 545 chromosome	171	171	100%	4e-49	100.00%	2935463	CP022908
S. aureus strain ER00503.3 chromosome	171	171	100%	4e-49	100.00%	2936779	CP030493
S. aureus strain UP_1452 chromosome	171	171	100%	4e-49	100.00%	2949972	CP047781
S. aureus strain ER02878.3 chromosome	171	171	100%	4e-49	100.00%	2968616	CP030713

Description	Max Score	Total Score	Query Cover	E. value	Per. Ident	Acc. Len	Accession
S. aureus strain ER11063.3 chromosome	171	171	100%	4e-49	100.00%	2969980	CP051914
S. aureus strain HC1335 chromosome	171	171	100%	4e-49	100.00%	2976370	CP012012
S. aureus strain Gv88 chromosome	171	171	100%	4e-49	100.00%	2988028	CP012018
S. aureus strain ER09403.3 chromosome	171	171	100%	4e-49	100.00%	3005577	CP051976
S. aureus strain Ryze chromosome	171	171	100%	4e-49	100.00%	3019690	CP113007
S. aureus strain Akali chromosome	171	171	100%	4e-49	100.00%	3022586	CP113032
S. aureus strain Zed chromosome	171	171	100%	4e-49	100.00%	3048932	CP113015
S. aureus strain JK3137 chromosome	171	171	100%	6e-49	100.00%	2955000	CP020960
S. aureus strain Sau26 chromosome	171	171	100%	6e-49	100.00%	2925858	CP141490
S. aureus strain E1 chromosome	171	171	100%	7e-49	98.78%	2931383	CP157976
S. aureus strain A1 chromosome	171	171	100%	7e-49	98.78%	2936542	CP140612
S. aureus strain E2 chromosome	171	171	100%	7e-49	98.78%	2944378	CP157977
S. aureus strain E3 chromosome	171	171	100%	7e-49	98.78%	2944807	CP157978
S. aureus strain C910 chromosome	171	171	100%	8e-49	98.78%	2775416	CP127543
S. aureus strain 30366_IOW744N4 chromosome	170	170	100%	1e-48	98.78%	2775300	CP069351
S. aureus strain TUM22702 chromosome	170	170	100%	1e-48	98.78%	2955722	CP150744
S. aureus strain TUM22702 chromosome	170	170	100%	1e-48	98.78%	2955727	CP162430
S. aureus strain TUM22701 chromosome	170	170	100%	1e-48	98.78%	2958459	CP150747
S. aureus strain TUM22701 chromosome	170	170	100%	1e-48	98.78%	2959317	CP162433
S. aureus strain O55 B118 chromosome	170	170	100%	1e-48	98.78%	2794042	CP038268
S. aureus strain BSN180 chromosome	170	170	100%	1e-48	98.78%	2813540	CP150881
S. aureus strain 17CS1042 chromosome	170	170	100%	1e-48	98.78%	2827946	CP138360
S. aureus strain ER04164.3 chromosome	170	170	100%	1e-48	98.78%	2837559	CP030542
S. aureus strain BSN85 chromosome	170	170	100%	1e-48	98.78%	2842199	CP157306
S. aureus CA-347	170	170	100%	1e-48	98.78%	2850503	CP006044
S. aureus strain ER04402.3 chromosome	170	170	100%	1e-48	98.78%	2853044	CP030505
S. aureus strain BSN25 chromosome	170	170	100%	1e-48	98.78%	2856082	CP158667
S. aureus strain ER03737.3 chromosome	170	170	100%	1e-48	98.78%	2857621	CP030641
S. aureus strain ER02919.3 chromosome	170	170	100%	1e-48	98.78%	2861613	CP030688
S. aureus strain BSN129 chromosome	170	170	100%	1e-48	98.78%	2862598	CP151822
S. aureus strain ER10234.3 chromosome	170	170	100%	1e-48	98.78%	2867130	CP051939
S. aureus strain ER10667.3 chromosome	170	170	100%	1e-48	98.78%	2871890	CP051929
S. aureus strain pt246 chromosome	170	170	100%	1e-48	98.78%	2888623	CP049454
S. aureus strain ER11327.3 chromosome	170	170	100%	1e-48	98.78%	2900855	CP051909
S. aureus strain BSN71 chromosome	170	170	100%	1e-48	98.78%	2901695	CP157302
S. aureus strain AR_474 chromosome	170	170	100%	1e-48	98.78%	2906506	CP030326
S. aureus subsp. aureus JH9	170	170	100%	1e-48	98.78%	2906700	CP000703
S. aureus strain pt192 chromosome	170	170	100%	1e-48	98.78%	2913317	CP049551
S. aureus strain 16439 chromosome	170	170	100%	1e-48	98.78%	2915632	CP043300
S. aureus strain ER09706.3 chromosome	170	170	100%	1e-48	98.78%	2916678	CP051961
S. aureus strain BSN188 chromosome	170	170	100%	1e-48	98.78%	2927162	CP156766
S. aureus strain BSN49 chromosome	170	170	100%	1e-48	98.78%	2931131	CP159794
S. aureus strain pt296 chromosome	170	170	100%	1e-48	98.78%	2932301	CP049379
S. aureus strain BSN48-2 chromosome	170	170	100%	1e-48	98.78%	2932403	CP157420
S. aureus strain ER02360.3 chromosome	170	170	100%	1e-48	98.78%	2936067	CP030393
S. aureus strain pt229 chromosome	170	170	100%	1e-48	98.78%	2937714	CP049485
S. aureus strain pt232 chromosome	170	170	100%	1e-48	98.78%	2938322	CP049991
S. aureus strain 16445 chromosome	170	170	100%	1e-48	98.78%	2940576	CP043302

Description	Max Score	Total Score	Query Cover	E. value	Per. Ident	Acc. Len	Accession
S. aureus strain BSN14RB chromosome	170	170	100%	1e-48	98.78%	2944325	CP080054
S. aureus strain BSN14R1 chromosome	170	170	100%	1e-48	98.78%	2944341	CP080053
S. aureus strain BSN14S3 chromosome	170	170	100%	1e-48	98.78%	2944342	CP080055
S. aureus strain BSN100 chromosome	170	170	100%	1e-48	98.78%	2944470	CP149463
S. aureus strain pt293 chromosome	170	170	100%	1e-48	98.78%	2950069	CP049381
S. aureus strain BSN207 chromosome	170	170	100%	1e-48	98.78%	2954066	CP158422
S. aureus strain 2395 USA500	170	170	100%	1e-48	98.78%	2955646	CP007499
S. aureus strain NRS120 chromosome	170	170	100%	1e-48	98.78%	2955890	CP026072
S. aureus strain CUBIST-16 chromosome	170	170	100%	1e-48	98.78%	2961621	CP168091
S. aureus strain BSN82 chromosome	170	170	100%	1e-48	98.78%	2964297	CP157308
S. aureus strain ER02836.3 chromosome	170	170	100%	1e-48	98.78%	2965519	CP030432
S. aureus strain BSN94 chromosome	170	170	100%	1e-48	98.78%	2967725	CP151423
S. aureus strain IVB6196 chromosome	169	169	100%	2e-48	98.78%	2808124	CP094772
S. aureus strain IVB6243 chromosome	169	169	100%	2e-48	98.78%	2813486	CP094752
S. aureus strain IVB6242 chromosome	169	169	100%	2e-48	98.78%	2813489	CP094755
S. aureus strain IVB6173 chromosome	169	169	100%	2e-48	98.78%	2785549	CP094780
S. aureus strain IVB6165 chromosome	169	169	100%	2e-48	98.78%	2785555	CP094793
S. aureus strain IVB6252 chromosome	169	169	100%	2e-48	98.78%	2785956	CP094743
S. aureus strain IVB6248 chromosome	169	169	100%	2e-48	98.78%	2786100	CP094750
S. aureus strain Dresden-275757 chromosome	169	169	100%	2e-48	98.78%	2789663	CP054876
S. aureus subsp. aureus strain GR2	169	169	100%	2e-48	98.78%	2792802	CP010402
S. aureus NCTC13435, chromosome : 1	169	169	100%	2e-48	98.78%	2797452	LN831036
S. aureus strain IVB6221 chromosome	169	169	100%	2e-48	98.78%	2809177	CP094763
S. aureus strain N12HSA28 chromosome	169	169	100%	2e-48	98.78%	2833771	CP091523
S. aureus subsp. aureus 11819-97	169	169	100%	2e-48	98.78%	2846546	CP003194
S. aureus strain ER01532.3 chromosome	169	169	100%	2e-48	98.78%	2876387	CP030695
S. aureus strain HPV107 chromosome	169	169	100%	2e-48	98.78%	2877716	CP026074
S. aureus strain WCUH29 chromosome	169	169	100%	2e-48	98.78%	2909934	CP039156
S. aureus strain CUBIST-25 chromosome	169	169	100%	2e-48	98.78%	2921731	CP168087
S. aureus strain 7-7 chromosome	169	169	100%	2e-48	98.78%	2737750	CP126626
S. aureus strain 33-40 chromosome	169	169	100%	2e-48	98.78%	2742607	CP126631
S. aureus strain 35-42 chromosome	169	169	100%	2e-48	98.78%	2742763	CP126629
S. aureus strain IVB6156 chromosome	169	169	100%	2e-48	98.78%	2853773	CP095116
S. aureus strain ER04648.3 chromosome	168	168	100%	6e-48	97.56%	2900707	CP052028
S. aureus strain GD705 chromosome	168	168	100%	6e-48	97.56%	2790096	CP019593
S. aureus strain MW2-16 chromosome	168	168	100%	7e-48	97.56%	2777737	CP151929
S. aureus strain C96 chromosome	168	168	100%	7e-48	97.56%	2793863	CP127773
S. aureus strain M37 chromosome	168	168	100%	7e-48	97.56%	2798511	CP071347
S. aureus strain C133 chromosome	168	168	100%	7e-48	97.56%	2800426	CP127702
S. aureus strain C179 chromosome	168	168	100%	7e-48	97.56%	2800426	CP127700
S. aureus strain M20 chromosome	168	168	100%	7e-48	97.56%	2806436	CP071348
S. aureus strain 950122 chromosome	168	168	100%	7e-48	97.56%	2809177	CP045441
S. aureus strain 55-100-016 chromosome	168	168	100%	7e-48	97.56%	2809746	CP076839
S. aureus strain USA400-0051 chromosome	168	168	100%	7e-48	97.56%	2811886	CP019574
S. aureus strain 2288 chromosome	168	168	100%	7e-48	97.56%	2814563	CP026646
S. aureus strain Mw2 chromosome	168	168	100%	7e-48	97.56%	2819334	CP026073
S. aureus strain MW2-47 chromosome	168	168	100%	7e-48	97.56%	2820174	CP151922
S. aureus strain MW2-S1 chromosome	168	168	100%	7e-48	97.56%	2820462	CP151930

Description	Max Score	Total Score	Query Cover	E. value	Per. Ident	Acc. Len	Accession
S. aureus subsp. aureus MW2 DNA	168	168	100%	7e-48	97.56%	2820462	BA000033
S. aureus strain MW2-45 chromosome	168	168	100%	7e-48	97.56%	2820473	CP151923
S. aureus strain MW2-48 chromosome	168	168	100%	7e-48	97.56%	2820507	CP151921
S. aureus strain MW2-25 chromosome	168	168	100%	7e-48	97.56%	2820516	CP151927
S. aureus strain MW2-35 chromosome	168	168	100%	7e-48	97.56%	2820538	CP151925
S. aureus strain MW2-23 chromosome	168	168	100%	7e-48	97.56%	2820541	CP151928
S. aureus strain MW2-32 chromosome	168	168	100%	7e-48	97.56%	2820554	CP151926
S. aureus strain MW2-42 chromosome	168	168	100%	7e-48	97.56%	2820590	CP151924
S. aureus strain FDAARGOS_43 chromosome	168	168	100%	7e-48	97.56%	2820631	CP026957
S. aureus strain MW2-50 chromosome	168	168	100%	7e-48	97.56%	2820698	CP151920
S. aureus strain LA-MRSA ST398, sequence	168	168	100%	7e-48	97.56%	2831848	CP013218
S. aureus strain ISU926 ST398 chromosome	168	168	100%	7e-48	97.56%	2833430	CP017091
S. aureus strain NZ15MR0322, chromosome: 1	168	168	100%	7e-48	97.56%	2839203	LT699704
S. aureus strain 14 chromosome	168	168	100%	7e-48	97.56%	2856550	CP094926
S. aureus strain 15 chromosome	168	168	100%	7e-48	97.56%	2856558	CP094925
S. aureus strain 18 chromosome	168	168	100%	7e-48	97.56%	2870995	CP094924
S. aureus strain 13 chromosome	168	168	100%	7e-48	97.56%	2872257	CP094927
S. aureus isolate 7_4623, chromosome: I	168	168	100%	7e-48	97.56%	2879411	LT992458
S. aureus isolate 8_LA_272, chromosome: I	168	168	100%	7e-48	97.56%	2891536	LT992461
S. aureus strain 111250134 chromosome	168	168	100%	7e-48	97.56%	2897518	CP045442
S. aureus 20_LA_415, chromosome: I	168	168	100%	7e-48	97.56%	2904888	LT992475
S. aureus E4_22S00627-1, chromosome: 1	168	168	100%	7e-48	97.56%	2922701	OZ059818
S. aureus E2_22S00623-1, chromosome: 1	168	168	100%	7e-48	97.56%	2927838	OZ059823
S. aureus H2_22S00625-1, chromosome: 1	168	168	100%	7e-48	97.56%	2927893	OZ059816
S. aureus E3_22S00626-1, chromosome: 1	168	168	100%	7e-48	97.56%	2927898	OZ059824
S. aureus strain C878 chromosome	167	167	100%	8e-48	98.78%	2760847	CP127544
S. aureus strain GP12 chromosome	167	167	100%	9e-48	98.78%	2754692	CP139866
S. aureus strain FDAARGOS_504 chromosome	167	167	100%	9e-48	98.78%	2894587	CP033865
S. aureus strain pt220 chromosome	167	167	100%	9e-48	98.78%	2903599	CP049503
S. aureus strain Sau6 chromosome	167	167	98%	1e-47	98.77%	2900088	CP141512
S. aureus strain RGB-095930 chromosome	167	167	98%	1e-47	98.77%	2904556	CP077098
S. aureus strain CC479-MSSA chromosome	167	167	100%	1e-47	97.56%	2739900	CP155058
S. aureus strain 24EBSta0529 chromosome	166	166	100%	3e-47	97.56%	2767774	CP166872
S. aureus strain B4-59C chromosome	166	166	100%	3e-47	97.56%	2781709	CP042153
S. aureus strain B2-15A chromosome	166	166	100%	3e-47	97.56%	2806828	CP042043
S. aureus strain B8-13D chromosome	166	166	100%	3e-47	97.56%	2807514	CP042107
S. aureus strain CUBIST-10 chromosome	166	166	100%	3e-47	97.56%	2816922	CP167764
S. aureus strain ch21 chromosome	166	166	100%	3e-47	97.56%	2822599	CP017804
S. aureus subsp. aureus ED98	166	166	100%	3e-47	97.56%	2824404	CP001781
S. aureus strain pt239 chromosome	166	166	100%	3e-47	97.56%	2833595	CP049467
S. aureus strain B3-17D chromosome	166	166	100%	3e-47	97.56%	2840146	CP042157
S. aureus strain N29CSA02 chromosome	166	166	100%	3e-47	97.56%	2855262	CP119341
S. aureus strain SA01 chromosome	166	166	100%	3e-47	97.56%	2883539	CP053075
S. aureus strain 2868B2 chromosome	166	166	100%	3e-47	97.56%	2884711	CP060141
S. aureus strain ER00551.3 chromosome	166	166	100%	3e-47	97.56%	2900663	CP030424
S. aureus strain ER04181.3 chromosome	166	166	100%	3e-47	97.56%	2907452	CP030547
S. aureus strain pt173 chromosome	166	166	100%	3e-47	97.56%	2907451	CP049580
S. aureus strain pt217 chromosome	166	166	100%	3e-47	97.56%	2907976	CP049510

Description	Max Score	Total Score	Query Cover	E. value	Per. Ident	Acc. Len	Accession
S. aureus strain NV_1 chromosome	166	166	100%	3e-47	97.56%	2939569	CP080249
S. aureus strain ch22 chromosome	166	166	100%	3e-47	97.56%	3074078	CP017807
S. aureus str. nan_175_F371_ch chromosome	166	166	98%	3e-47	97.53%	2793849	CP066492
S. aureus strain 7062 chromosome	166	166	100%	3e-47	97.56%	2870460	CP118810
S. aureus strain CC479-MSSA chromosome	166	166	100%	4e-47	97.56%	2736525	CP155061
S. aureus strain ncr_155_F133 chromosome	163	163	98%	3e-46	96.30%	2820908	CP066488
S. aureus strain 22-042 chromosome	149	149	100%	2e-41	85.37%	2806604	CP128387
S. aureus strain 21-074 chromosome	149	149	100%	2e-41	85.37%	2806618	CP128388
S. aureus strain RIVM_M047065 chromosome	140	140	89%	5e-38	91.78%	2806671	CP096539
S. aureus strain BSN142 chromosome	140	140	89%	5e-38	91.78%	2808584	CP151289
S. aureus isol. E1_22S00622-1, chromosome	140	140	89%	5e-38	91.78%	2927810	OZ059820
S. aureus strain MRSA107 chromosome	139	139	89%	6e-38	91.78%	3095697	CP018629
S. aureus strain Sau38 chromosome	100	181	100%	6e-38	100.00%	2798910	CP141477
S. aureus strain Syndra chromosome	100	177	100%	5e-37	100.00%	2921835	CP113027
S. aureus strain Be62 chromosome	123	177	100%	6e-37	98.31%	2998480	CP012013
S. aureus strain 18-22 chromosome	99.0	176	100%	9e-37	97.87%	2741653	CP126630
S. aureus strain 1824 chromosome	120	173	100%	1e-35	94.92%	2871168	CP094923
S. aureus strain Taliyah chromosome	100	156	100%	9e-31	100.00%	3043269	CP113018
S. aureus strain Ahri chromosome	94.4	151	96%	4e-29	100.00%	2923858	CP113049

*The search was performed with the use of BlastP, using the complete genomic sequences deposited in GenBank (accessed 2024-10-23) and the ASZ22RN Cro protein sequence as a query.

Table S11. Recognition sites for various staphylococcal RM systems in ASZ22RN and pLKA18 DNA

Type of RM system	Enzyme or Clonal Complex	Recognition Sequence	Number of recognition sites in ASZ22RN DNA	Number of recognition sites in pLKA18 DNA
I	CC1-1	CCAY(N)$_5$TTAA[a]	6	0
	CC1-2 (CC8-2)	CCAY(N)$_6$TGT[a#]	10	2
	CC5-1 (CC8-1, CC8-2)	AGG(N)$_5$GAT[a#]	18	3
	CC5-2	CCAY(N)$_6$GTA[a#]	6	0
	CC15-1	CAAC(N)$_5$RTGA	4	1
	CC22-1	AGG(N)$_6$TGAR[a]	7	0
	CC30-1	GWAG(N)$_5$GAT[#]	10	2
	CC30-2	GGA(N)$_7$TCG[#]	5	2
	CC-45	GWAG(N)$_6$TAAA[a#]	7	0
	CC59-1	GGA(N)$_6$RTGT	5	1
	CC97-1	CCAY(N)$_6$RTC	12	1
	CC398-1	ACC(N)$_5$RTGA[#]	7	1
II	Sau3A	GATC	47	31
	Sau96I	GGNCC	8	13
IV	SauUSI	S5mCNGS*	819	447

[#] Recognition sites for the R-M systems of *S. aureus* strains that served as plasmid recipients in ASZ22RN-mediated transduction.
[a]Recognition sites for the R-M systems of some *S. aureus* strains that were productively infected with ASZ22RN
*Cut only when methylated

Supplementary Figures

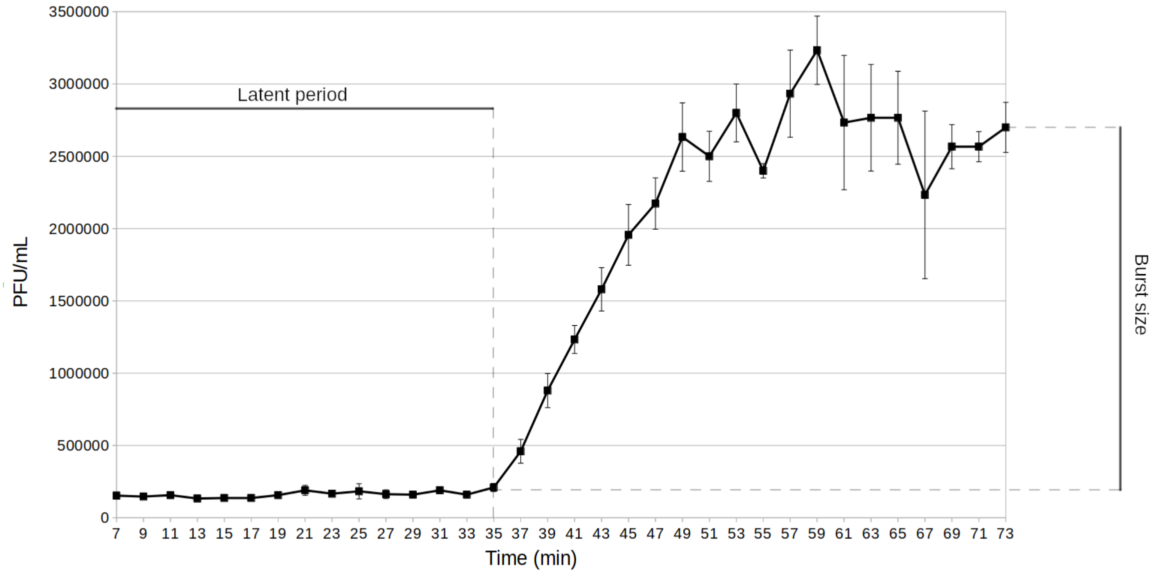

Figure S1. One step growth curve of phage ASZ22RN in RN4220 cells.

A

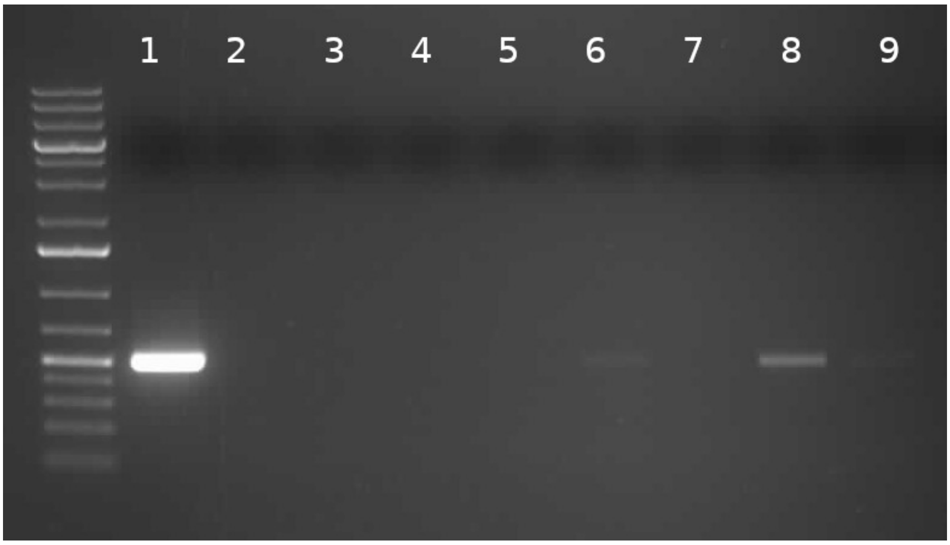

B

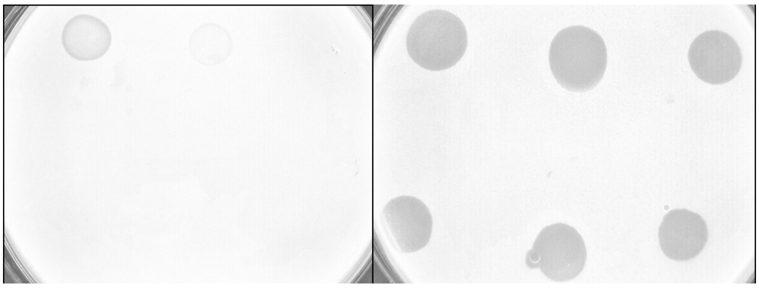

Figure S2. Detection of RN4220 cells lysogenized with phage ASZ22RN and the resistance of lysogen to superinfection with ASZ22RN. **A.** Amplicons obtained in colony PCR with DNA of potential ASZ22RN lysogens as a template (lane 2-9) and primers 5'-TTGATTAAATTAACACCG-AAGCAAGAAAAGTTTGTATTA and 5'-TTATTCATTGACGATCACTTCCGTTATTGC, specific for ASZ22RN DNA. The leftmost lane represents 1 Kb Plus Ladder (Thermo Fischer Scientific Baltics, Vilnius, Lithuania), lane 1 represents positive control - ASZ22RN DNA. **B.** The difference in the sensitivity of wild-type RN4220 strain and its lysogen to infection with phage ASZ22RN. Serially diluted lysates containing phage ASZ22RN (10^{10} pfu/ml of initial phage titer) were spotted on the layer of *S. aureus* RN4220 cells containing ASZ22RN prophage (left) and *S. aureus* RN4220 cells without a prophage (right). The colors on the images were reversed and desaturated to increase the visibility of lysis.

A

```
ASZ22RN gp4 145  KHRDEVLKKIRMHITQGIMSGEGYSKIAKAIRDDVGMSKAQSLRVARTEAGRAMSQAGLDSAMVAKDNGL  214
                 +H +E +K +R H+ +    G    K A AI+            +  TE+ R  +Q+ +DS       G
80alfa gp44 202  RHPNEYVKDMRKHLNK--FEGTARQKTA-AIKS-----------LLYTESARVHAQSSIDSMKEISPEGY  257

ASZ22RN gp4 205  KMKKRWHATKDTRTRDTHRHLDGESVEIDQNFKSSGCVGQAPKLFIGVNSAKENINCR--CKLL  276 of 317
                  M   + A  D RT    + L+GE             + +     IGVN    +INCR  C LL
80alfa gp44 248  YM---YIAKIDNRTTKVCKGLNGE-------------IFKVKDAKIGVNFYPMHINCRSDCALL  305 of 331
```

B

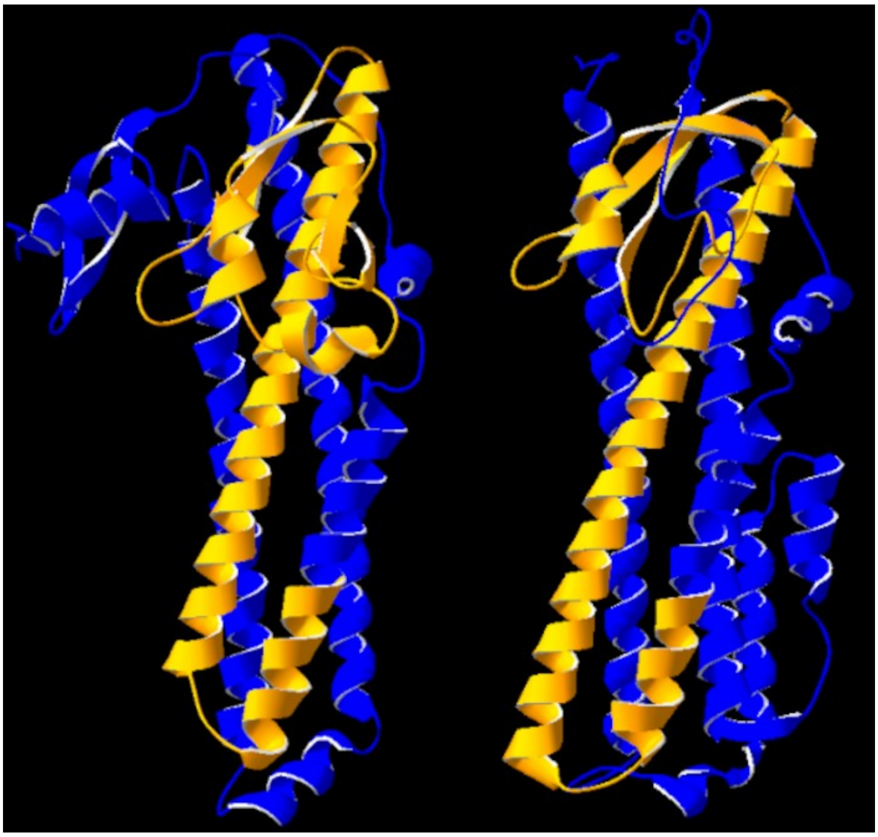

Figure. S3. Sequence and structure similarities between phage ASZ22RN gp4 and phage 80alfa gp44. **A.** Alignment of the predicted MuF domain region of gp4 (WCS65156) with the Mu domain region of gp44 (YP_001285358). The amino acid residues assigned to the MuF domains are bolded. The regions of MuF domains were predicted with the use of InterPro (7) at its web site (https://www.ebi.ac.uk/interpro/) **B.** Structure prediction of phage ASZ22RN gp4 (left) and phage 80alpha gp44 (right). The MuF domains are in yellow. Structures were predicted with the use of AlphaFold 3 (8) and visualized with the use of Swiss-PdbViewer (9).

ASZ22RN *attP*

```
        -140      -130      -120      -110     -100        -90       -80       -70      -60      -50
TCAAACGTACTCTCATTTATATACTGATAAAAAACATCAAGCTATGAGCATATTTGATTAAACGGTATCAAATTGGTATCAAATAACAATTAAGGAGTTT

        -40       -30       -20       -10       -1+1       10        20        30        40       50
ATAAAATGCGTAATAACAAGCCTAAAATAAGTATTCAAAACGACCCATG|GGAAGTGAAATTTATATACATTTAAATTTCATGAGACAATAAACGTTGAT
  :: :     ::    ::::      : :: ::: : :: :: ::::: :::::   :          :   ::  :  :  :   : :  : ::
TGAATAGCGTCAAATTGAAGCAACCTGTTAGCATTTACAATGATCCATG|GGAAGCATATAACGATGTTAAAGAACATGGCCAATTAACTTTAAGTAACA

         60        70        80        90      100        110       120       130      140      150
TTAATGCGTTTTTTGCCTTTTTTATTTTCCTTATTTTTTCTGTTTTACAACAAAATGGTATCAAAAATGGTATCATTTGTAGTTATTTTAGCTTCACATA
```

Figure S4. The sequence of phage ASZ22RN *attP* region and its alignment with the *attB* of 4472/08 strain (shown in blue). The core regions of *attB* and *attB* are underlined, with the recombination sites indicated with "|". The sequence is numbered from the center of the core. The regions of tandem repeats representing integrase-binding sites identical to those of phage phi11 are highlighted in grey (10).

A.

```
S. aureus YfkA N-terminus                1..MNQIERRKFKTMNSVKLKQPVSIYNDPWE
                                                             |  +|| |||||
Alternative ASZ22RN provided N-terminus               1..MRNNKPKISIQNDPWE
```

B.

```
                                                 -35                        -10
26080..TGAGGCATTCTAACATTACAGAAACAATTCAAACGTACTCTCATTTATATACTGATAAAAAACATCAAGC
TATGAGCATATTTGATTAAACGGTATCAAATTGGTATCAAATAACAATTAAGGAGTTTATAAAATGCGTA..26219
```

Figure S5. Coding potential of the leftmost end of the ASZ22RN prophage DNA. **A.** Alignment of the amino acid sequence of the N-terminus of *S. aureus* 4472/08 YfkA protein with the predicted alternative N-terminus of YfkA provided by the ASZ22RN prophage in lysogens. **B.** The DNA sequence preceding the start codon for the predicted ASZ22RN-encoded alternative YfkA N-terminus. The beginning of the alternative *yfkA* gene is in bold. Nucleotide residues encoding predicted Shine-Dalgarno sequence in mRNA and complementary to the 3'-end of staphylococcal 16S rRNA (GenBank acc. no. NR_118997.2) are underlined. The -35 and -10 regions of predicted σ^{70} promoter for RNA polymerase identified with the use of Promoter Hunter (11, 12) at its website (http://www.phisite.org/main/index.php?nav=tools&nav_sel=hunter) are highlighted in grey. The ASZ22RN sequence coordinates are as in GenBank file ON513432.1.

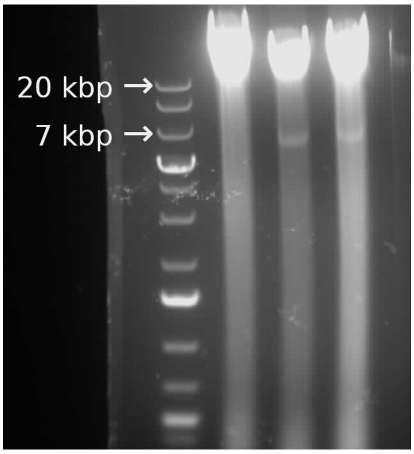

Figure. S6. Comparison of total DNA from the wild-type RN4220 cells (lane 2), and cells electrotransformed with the pLKA18 plasmid (lane 3) or transduced with the pLKA18 plasmid with phage ASZ22RN (lane 4). DNA size marker is shown in lane 1 (Thermo Scientific GeneRuler 1 kb Plus DNA Ladder). Total DNA was isolated with the use of QIAGEN Genomic-tip 100/G. Overnight cultures of cells for DNA isolation were grown in LB medium (supplemented with chloramphenicol 20 µl/ml where needed) refreshed by 50x dilution in 50 ml of fresh medium and incubated for 16 h at 37ºC. Cells were harvested by centrifugation at 6000 x g for 15 min. Total DNA was isolated with the use of QIAGEN Genomic-tip 100/G according to the manufacturer's protocol. Before DNA isolation, the cells were pretreated with lysostaphin (0.1 mg/ml), resuspended in buffer P1 and incubated for ~40 min. at 37ºC. DNA was separated electrophoretically in 0.7% agarose gel.

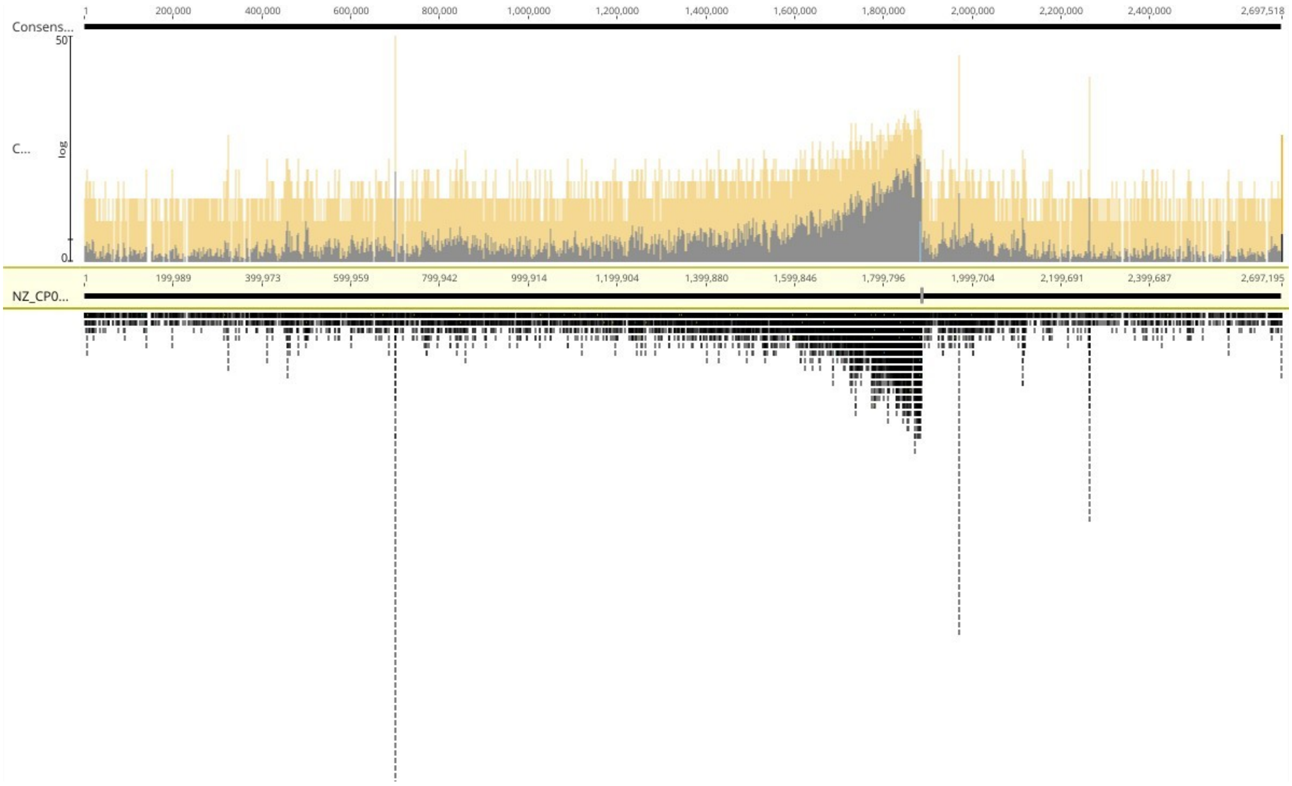

Figure S7. Representation of various genomic fragments of *S. aureus* RN4220 strain in transducing particles of phage ASZ22RN. Genome coordinates are above the black line representing the DNA of RN4220 strain. The gray bar on the line representing genomic DNA indicates the ASZ22RN attachment site. Sequence reads were aligned to the DNA sequence of RN4220 strain (GenBank Acc. No. NZ_CP076105) with the use of Geneious Prime using default parameters.

Supplementary References

1. Asheshov EH. 1969. The genetics of penicillinase production in *Staphylococcus aureus* strain ps 80. *Microbiology* 59:289–301.
2. Nair D, Memmi G, Hernandez D, Bard J, Beaume M, Gill S, Francois P, Cheung AL. 2011. Whole-genome sequencing of *Staphylococcus aureus* strain RN4220, a key laboratory strain used in virulence research, identifies mutations that affect not only virulence factors but also the fitness of the strain. *J Bacteriol* 193:2332–2335.
3. Głowacka-Rutkowska A, Gozdek A, Empel J, Gawor J, Żuchniewicz K, Kozińska A, Dębski J, Gromadka R, Łobocka M. 2019. The ability of lytic staphylococcal *podovirus* vB_SauP_phiAGO1. 3 to coexist in equilibrium with its host facilitates the selection of host mutants of attenuated virulence but does not preclude the phage antistaphylococcal activity in a nematode infection model. *Frontiers in Microbiology* 9:3227.
4. Devriese LA, Hájek V, Oeding P, Meyer SA, Schleifer KH. 1978. *Staphylococcus hyicus* (Sompolinsky 1953) comb. nov. and *Staphylococcus hyicus* subsp. *chromogenes* subsp. nov. *International Journal of Systematic and Evolutionary Microbiology* 28:482–490.
5. Freney J, Brun Y, Bes M, Meugnier H, Grimont F, Grimont PAD, Nervi C, Fleurette J. 1988. *Staphylococcus lugdunensis* sp. nov. and *Staphylococcus schleiferi* sp. nov., Two species from Human Clinical Specimens. *International Journal of Systematic and Evolutionary Microbiology* 38:168–172.
6. Kahánková J, Pantůček R, Goerke C, Růžičková V, Holochová P, Doškař J. 2010. Multilocus PCR typing strategy for differentiation of *Staphylococcus aureus* siphoviruses reflecting their modular genome structure. *Environmental Microbiology* 12:2527-38.

7. Blum M, Andreeva A, Florentino LC, Chuguransky SR, Grego T, Hobbs E, Pinto BL, Orr A, Paysan-Lafosse T, Ponamareva I, Salazar GA, Bordin N, Bork P, Bridge A, Colwell L, Gough J, Haft DH, Letunic I, Llinares-López F, Marchler-Bauer A, Meng-Papaxanthos L, Mi H, Natale DA, Orengo CA, Pandurangan AP, Piovesan D, Rivoire C, Sigrist CJA, Thanki N, Thibaud-Nissen F, Thomas PD, Tosatto SCE, Wu CH, Bateman A. 2025. InterPro: the protein sequence classification resource in 2025. *Nucleic Acids Research* 6;53(D1):D444-D456.
8. Abramson J, Adler J, Dunger J, Evans R, Green T, Pritzel A, Ronneberger O, Willmore L, Ballard AJ, Bambrick J, Bodenstein SW, Evans DA, Hung CC, O'Neill M, Reiman D, Tunyasuvunakool K, Wu Z, Žemgulytė A, Arvaniti E, Beattie C, Bertolli O, Bridgland A, Cherepanov A, Congreve M, Cowen-Rivers AI, Cowie A, Figurnov M, Fuchs FB, Gladman H, Jain R, Khan YA, Low CMR, Perlin K, Potapenko A, Savy P, Singh S, Stecula A, Thillaisundaram A, Tong C, Yakneen S, Zhong ED, Zielinski M, Žídek A, Bapst V, Kohli P, Jaderberg M, Hassabis D, Jumper JM. 2024. Accurate structure prediction of biomolecular interactions with AlphaFold 3. *Nature*. 630:493-500.
9. Guex N, Peitsch MC. 1997. SWISS-MODEL and the Swiss-PdbViewer: an environment for comparative protein modeling. *Electrophoresis* 18:2714-23.
10. Lee CY, Iandolo JJ. Structural analysis of staphylococcal bacteriophage phi 11 attachment sites. 1988. *Journal of Bacteriology* 170:2409-11.
11. Klucar, L., Stano, M., Hajduk, M. 2010. phiSITE: Database of Gene Regulation in Bacteriophages. *Nucleic Acids Research* 38 (Database Issue): D366-D370.

12. Stano, M., Klucar, L. 2011. phiGENOME: An integrative navigation throughout bacteriophage genomes. *Genomics* 98:376-380.
